# Biofabricated Alginate Hydrogels to Study Prostate Tumoral Microenvironments In Vitro

**DOI:** 10.1021/acsomega.5c13436

**Published:** 2026-04-29

**Authors:** Khalsa Al-Husaini, Eugenia Spessot, Esther Baena, Marco Domingos, Annalisa Tirella

**Affiliations:** † Division of Pharmacy and Optometry, Medicine and Health, The University of Manchester, M13 9PL Manchester, U.K.; ‡ Biology Department, College of Science, 98816 Sultan Qaboos University, 123 Muscat, Oman; § Department of Industrial Engineering, Biotech Center for Biomedical Technologies, 19034 University of Trento, 38122 Trento, Italy; ∥ Cancer Research UK Manchester Institute, The University of Manchester, M13 9PL Manchester, U.K.; ⊥ Department of Mechanical and Aerospace Engineering, School of Engineering, Faculty of Science and Engineering & Henry Royce Institute, The University of Manchester, M13 9PL Manchester, U.K.

## Abstract

Engineered three-dimensional (3D) in vitro models are essential for recapitulating the human tumor microenvironment (TME) and deciphering the complex cell–material interactions driving cancer progression. This study presents the development of a prostate-specific bioprinted model designed to mimic both the biomechanical (stiffness) and biochemical (laminin-enriched) traits of the prostate cancer (PCa) extracellular matrix (ECM). We synthesized functionalized alginate hydrogels modified with laminin-mimetic peptides (IKVAV, AG73) and tuned their mechanical properties (2–20 kPa) to match the transition from healthy tissue to advanced/metastatic disease. Functionalized alginate hydrogel precursors were compatible with extrusion-based bioprinting and used to replicate TME heterogeneity by 3D bioprinting in vitro models containing PC-3 cells and cancer-associated fibroblasts (CAFs). PC-3 cells cocultured with cancer-associated fibroblasts (CAFs) within these hydrogels supported high cell viability and proliferation. Notably, phenotypic analysis revealed that stiffer, laminin-enriched matrices significantly upregulated the expression of CD44 and the epithelial-to-mesenchymal transition (EMT) marker vimentin in PC-3 cells. Interestingly, these matrix-driven effects were dominant, independent of the CAF presence within the observed window. This work establishes a robust, scalable biofabrication strategy for generating TME-mimetic models, offering a valuable tool for future studies in screening TME-targeting therapies and investigating the mechanobiology of PCa progression.

## Graphical abstract

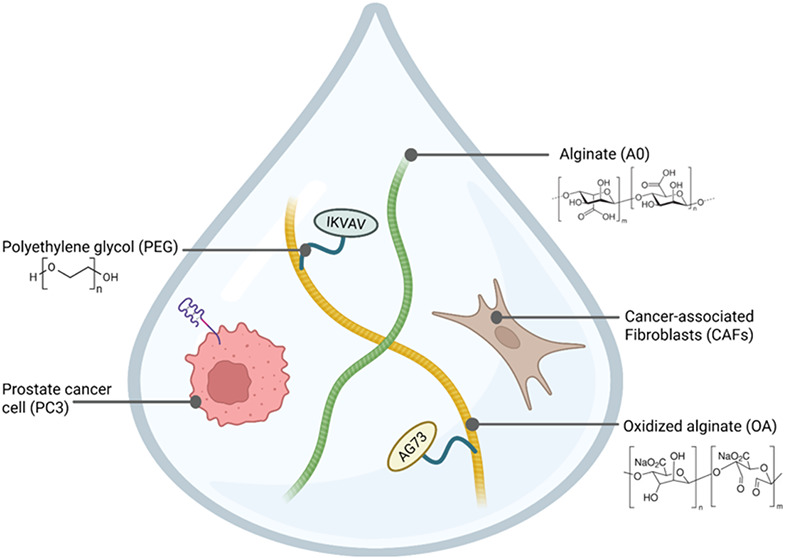

## 1 Introduction

Prostate cancer (PCa) is one of the most commonly diagnosed malignancies and a leading cause of cancer-related deaths in men globally.^1^ As in other solid cancers, variations of the composition of the tissue-specific extracellular matrix (ECM) by different cell types (e.g., cancer cells, fibroblasts, macrophages) directs tumor progression, metastasis, and drug resistance, typically associated with poor prognosis.^2,3^ ECM stiffening and remodeling during PCa progression is often driven by cancer-associated fibroblasts (CAFs),^4^ such ECM alterations cause phenotypic variation of cancer cells toward more invasive, proliferative, and drug-resistant ones. Despite knowing that ECM stiffening during PCa progression modulates cells’ phenotype and metastatic potential, specific cell–ECM interactions remain poorly understood, limiting the development of effective therapeutic strategies.^5,6^ Among the many ECM components present in healthy and tumor prostate tissues, altered laminin profiles were recorded during PCa progression and linked to different outcomes in patients with prostate adenocarcinoma.^7^ Laminins are high-molecular-weight heterotrimeric glycoproteins composed of three subunits (i.e., α-, β-, and γ-subunits) and are the main constituents of the basement membrane with various adhesive, stimulatory, and biological functions.^8^ Alterations of both laminin and stiffness of prostate-specific ECM directly correlate with the aggressiveness of PCa cell phenotypes, mainly dictated by integrin-mediated interactions, causing PCa cell transformation, proliferation, stemness, and invasion.^8,9^ The clinical correlation of laminin-α expression in its different forms (e.g., α1, α3, α5) in prostate ECM is associated with PCa progression and metastasis,^8,10–12^ with laminin-111 (i.e., α1, β1, and γ1 chains), the ligand for α6 integrins, being detected in elevated levels in grade II and III prostate tumors.^13^ Despite poor knowledge on the correlation between laminin expression in the TME and prognosis scores in clinical settings (e.g., Gleason’s score), understanding differences in laminin expressions in different PCa ECM (e.g., androgen-dependent, castration-resistant prostate cancer) could unveil integrin-mediated interactions between cancer cells and ECM; hence, the design of microenvironments able to mimic alterations of laminin and stiffness is paramount to predict PCa cells’ clinical profiles.

To overcome the inherent limitations of 2D culture and accurately capture the multifactorial nature of PCa progression, engineered three-dimensional (3D) in vitro models are used to reproduce healthy prostate and altered PCa microenvironments^14,15^ and to provide an alternative to PCa spheroids.^16^ Successful 3D tumor in vitro models must integrate the primary cellular constituents of the TME, such as PCa cells (e.g., PC-3) and CAFs, which are known to drive ECM remodeling and stiffening.

For this reason, we decided to use hydrogels to support cell growth that allows us to mimic disease-specific prostate TME more effectively in its biophysical properties.^17^

Alginate was selected due to its biocompatibility and ability to tune stiffness by easy ionic cross-linking, making it widely used in many 3D in vitro models.^18–21^ Alginate-based biomaterial inks are also widely used in extrusion-based bioprinting (EBB) for the design and fabrication of 3D in vitro models that replicate the spatial organization of different microenvironments, enabling precise patterning of PCa cells and CAFs and controlling hydrogel properties as an ECM-mimicking scaffolding material.^22,23^

The mechanical properties of prostate-specific alginate hydrogels were controlled by varying both alginate and cross-linker (CaCl_2_) concentration.^24,25^ Based on our previous studies,^26–28^ oxidized alginate (OA) was herein used to covalently link laminin-like peptides (e.g., IKVAV or AG73, which mimic binding sites for integrins like α6β1 ^12^) using bifunctional polyethylene glycol (PEG), as previously shown,^29^ to promote specific PCa cell–material interactions. Based on our previous study,^26^ we used OA with a 50% degree of oxidation to achieve a suitable laminin-mimicking peptide concentration crucial for studying how PCa cells exploit laminin-rich components for key events like migration and invasion.^13,30^

To validate the use of engineered 3D in vitro models, their ability to faithfully recapitulate malignant processes like metastasis and to study epithelial-to-mesenchymal transition (EMT) was assessed. Epithelial (E-cadherin) and mesenchymal (vimentin) markers were monitored with cancer stem-like markers (e.g., CD44) as demonstrators to predict prostate cancer (PCa) metastatic phenotype. Specifically, based on the clinical evidence of the role of stiffness in cancer progression^9^ and our previous findings on breast cancer cells,^31,32^ we evaluated how stiffness and laminin variations in engineered hydrogels impacted PC-3 cells’ phenotypes (EMT markers and CD44) and their correlation to their migratory and invasive phenotype in the presence or absence of CAFs. The mechanical properties of hydrogels were controlled using different concentrations of the cross-linker (CaCl_2_), with compression tests showing Young’s modulus in the range of PCa (i.e., 1–20 kPa), as reported in the literature.^33–37^ Rheological tests confirmed the printability of prostate-specific alginate biomaterial inks, and 3D printed in vitro models encapsulating PC-3 cells and CAFs were fabricated via EBB. PC-3 cells showed good viability and proliferation in the designed hydrogel up to 14 days, with significantly enhanced proliferation in softer and laminin-rich hydrogels (E = 3.0 ± 0.5 kPa, **A1-P**) compared to stiffer and not functionalized hydrogels (E = 12.6 ± 1.3 kPa, hydrogel **A3**). Dysregulated CD44 expression with the increase of vimentin, linked to the loss of CD44v isoforms and reduced E-cadherin in PC-3 cells, was observed, indicative of invasive and metastatic traits of PCa cells.

Current in vitro platforms typically fail to decouple biomechanical stiffness from biochemical composition, preventing a clear understanding of specific TME drivers. To address this limitation, this study presents a technological platform combining laminin-enriched functionalized alginates to bioprint 3D PCa in vitro models as a new approach methodology (NAM) to study cancer cell biology and behavior. We hypothesize that functionalized alginate hydrogels, decoupling the presence of laminin-mimicking peptide sequences and stiffness, can more accurately recapitulate the aggressive phenotype of PC-3 cells and unveil the interplay between cell–material interactions and the presence of CAFs on aggressive PCa phenotypes in vitro.

This model recapitulates key physicochemical and biological cues of the TME, aligning with recent guidelines for NAMs to evaluate human cancer progression in vitro. While the specific conditions tested herein were defined by the current knowledge of the PCa ECM, our manufacturing pipeline is designed for scalability and reproducibility. Consequently, this system offers a versatile platform to screen a broad range of matrix properties, paving the way for new insights into patient-specific PCa progression.

## 2 Materials and Methods

### 2.1 Materials

Sodium alginate (71238), sodium periodate (NaIO_4_, S1878), 3-(trimethylsilyl)-2,2,3,3-tetradeuteropropionic acid (TMSP-d4, A14489.03), deuterium oxide (D2O, 7789-20-0), N-(2-Hydroxyethyl)­piperazine-N′-(2-ethanesulfonic acid) (HEPES, H4034), gelatin type A (G1890), Pluronic F127 solution (P2443), nutrient mixture F-12 Ham medium (N6658), fetal bovine serum (FBS, F9665), 4% paraformaldehyde (PFA, 1004968350), l-glutamine (G751), sodium bicarbonate (NaHCO_3_, S8761), phosphate buffer solution (PBS, D1408), EMEM (M4655), sodium pyruvate (P5280), agarose (type 1, low EEO, A6013), Triton X (648465), saponin blocking buffer (47036), AccumaxTM (A7089), and fibronectin (F2006) were all ordered from Sigma–Aldrich. 5,5′-Dithio-bis­(2-nitrobenzoic acid) (DTNB, 44889), cysteine hydrochloride monohydrate (44889), sodium chloride (NaCl, Fisher, 7647-14-5), calcium chloride (CaCl2, C/1400/53), sodium hydroxide (NaOH, 12963614), Live/Dead Kit (calcein AM and ethidium homodimer (EthD-1), L3224), phalloidin Alexa Fluor 488 (A12379), and 4′-6-diamidino-2-phenylindole (DAPI, D1306) were all purchased from Thermo Fischer Scientific. Thiol-terminated laminin-like peptides (IKVAV and AG73 custom-made) were purchased from GenScript. Modified polyethylene glycol (H_2_N-PEG-Mal, PBH-943, MW: 5 kDa) was purchased from Creative PEGworks. A Deep Blue Cell Viability Kit (424701) was purchased from BioLegend. Cytopainter (ab138893) was purchased from Abcam. Puromycin (A11138–03) and cell dissociation buffer (13151014) were purchased from Gibco. The collagen hydrogel precursor (50201) was purchased from Ibidi.

### 2.2 Functionalization of Alginate

#### 2.2.1 Preparation of Oxidized Alginate

Oxidized alginate (OA) was prepared following our protocol, as reported in our previous studies, and targeting a 50% degree of oxidation (DO).^26,27^ Briefly, 8 g of sodium alginate (A_0_) was dissolved in 160 mL of deionized water (dH_2_O) at RT overnight with mechanical stirring (400 rpm) using a tornado parallel reactor (RZR 2020, Heidolph, Germany), and then 40 mL of 0.5 M NaIO_4_ (aq.) was gently added into the stirring alginate solution. The oxidation reaction was performed with continuous mechanical stirring (400 rpm) at RT for 6 h; then, the obtained OA_50_ solution (aq.) was purified by dialysis (Ultracel 3 kDa, PLBC07610) against dH_2_O at RT and in the dark to prevent further OA_50_ hydrolysis, changing the dH_2_O every day until completion of the purification process (i.e., dH_2_O conductivity <8 μS/cm). OA_50_ solution (aq) was freeze-dried (Chaist α 2–4 LSC; 0.01 mbar, −80 °C) until completely dry. The obtained OA_50_ powder was stored in the dark at RT and used for 18 months.

#### 2.2.2 Prostate-Specific Laminin-Enriched Alginates

Prostate-mimicking alginates were functionalized including laminin-111 sequences to better mimic prostate ECM via the linear heterobifunctional PEG (MAL-PEG- NH_2_), used to link peptides to OA_50_ via the maleimide–thiol Michael-type reaction.^38^ Thiol-terminated IKVAV (IKVAV-SH) was used as a model peptide to assess alginate functionalization. A 1:1 molar ratio of MAL-PEG- NH_2_ and IKVAV-SH were allowed to react with constant stirring under continuous flow of argon gas (3 h, RT). The obtained PEG–peptide conjugate product (IKVAV-PEG-NH_2_) was freeze-dried (0.01 mbar, −80 °C) and then linked to OA_50_ via a Schiff base reaction between the aldehyde groups (OA_50_) and the primary amino groups present on the PEG–peptide compound (Figure 2A). To assess the efficiency of the reaction, a 4% w/v OA_50_ solution in HBS (pH 8.5) was used, varying the initial concentration of IKVAV-PEG-NH_2_ (100 and 200 μM) with constant stirring (24 h, RT). The obtained solution was purified by dialysis (Spectra-Por MWCO 3.5–5 kDa, Z726273, Sigma–Aldrich, UK) against dH_2_O at RT until conductivity was <8 μS/cm. The obtained functionalized alginate (OA_50_-PEG-IKVAV) was freeze-dried and stored until use. For cell culture studies, two α-laminin thiol-terminated mimicking peptides were selected (IKVAV-SH, AG73-SH) and used in this study to form PEG–peptide conjugates, as reported in other studies,^38^ and then linked to OA_50_ to obtain alginate hydrogels enriched in laminin-mimicking domains.

### 2.3 Chemical Characterization of Prostate-Specific Alginates

#### 2.3.1 PEG-IKVAV Coupling Efficiency: Ellman’s Reagent

Ellman’s reagent (DTNB) was used to quantify MAL-PEG- NH_2_ and thiol-terminated peptide coupling efficiency. MAL-PEG- NH_2_ (aq) and IKVAV-SH (aq) were mixed and incubated at a 1:1 molar ratio (i.e., 500 μM) under argon gas with constant stirring at RT for 90 and 270 min. Samples were then mixed with DTNB following the manufacturer’s instructions. Absorbance was measured at 412 nm using a plate reader spectrophotometer (BioTek, Synergy 2, NorthStar Scientific Ltd.). A standard calibration curve using cysteine hydrochloride monohydrate (aq.) in the molar range of 0.25–1.5 mM quantified the unreacted peptide. The experiments were performed in triplicate (*n* = 3) and for *N* = 3 independent experiments.

#### 2.3.2 ^**1**^H NMR Spectroscopy

^1^H NMR was used to characterize alginate functionalization and to obtain the degree of functionalization (DF) using a Bruker Avance III-500 MHz NMR spectrometer, at 25 °C with a 90° pulse proton angle, a relaxation delay of 2 s, and an acquisition time of 4.096 s, with total scans of 128 for each sample. A_0_, OA_50_, and OA_50_-PEG-IKVAV samples were hydrated in 1 mL of deuterium oxide (D_2_O) at a concentration of 10 mg/mL (2 min, 50 °C). MAL-PEG-NH_2_, IKVAV-SH, and IKVAV-PEG-NH_2_ were hydrated in 600 μL in D_2_O at a concentration of 5 mg/mL at RT. Specifically, the DF was assessed using a 4% w/v OA_50_ solution (aq.) varying the concentration of MAL-PEG-NH_2_ (i.e., 100 μM, 200 μM), allowing the reaction for 24 h at RT. TMSP-d4 was used as an internal NMR standard to reference and quantify the coupling efficiency. Prior to acquisition, all samples were freeze-dried (0.01 mbar, −80 °C) until completely dry and then redissolved in D_2_O. Spectra were analyzed by TopSpin software (Bruker, 4.1.0).

### 2.4 Hydrogel Mechanical and Physical Properties

#### 2.4.1 Sample Preparation

##### 2.4.1.1 Hydrogel Precursor Solutions

Alginate and gelatin solutions (aq) were dissolved in HBS at RT and 37 °C, respectively, at concentrations reported in Table 1. Alginate and gelatin solutions were sterile-filtered using a 0.22 μm PES filter (Millex GP) and a 0.45 μm PVDF filter (Millex HV), respectively. Sterile solutions were gently mixed, paying attention not to generate air bubbles and obtaining biomaterial inks **A** (A_0_/gelatin/OA_50_) and **A-P** (A_0_/gelatin/OA_50_-PEG-peptides).

**Table 1:** Composition of Prostate-Specific Alginate Precursor Solutions and Hydrogels^a^

sample ID	A_0_ (% w/v)	OA_50_ (% w/v)	OA_50_-PEG-peptide (% w/v)	G (% w/v)	CaCl_2_ (μM)
**A**	1	1	0	3	0
**A1**	1	1	0	3	100
**A3**	1	1	0	3	300
**A-P**	1	0	1	3	0
**A1-P**	1	0	1	3	100
**A3-P**	1	0	1	3	300

a For hydrogel preparation, precursor solutions with unmodified alginate (A_0_), oxidized alginate (OA_50_), modified alginate (OA_50_-PEG-peptide), and gelatin (G) were mixed and then cross-linked with CaCl_2_ solutions (aq.). Concentrations reported in the table are the final ones for each alginate-based formulation.

##### 2.4.1.2 Hydrogel Samples

Cylindrical alginate-based hydrogels (Table 1) were prepared by adding a known volume of precursor solutions (aq.) in a cylindrical mold (i.e., 8 mm diameter), previously treated with a sterile 3% w/w Pluronic F127 solution (aq.) for easy removal of formed hydrogels.^27^ A first incubation (1 h, 4 °C) was set, allowing gelatin sol–gel transition to rapidly fix the hydrogels’ shape, followed by incubation, allowing for Schiff base formation. CaCl_2_ solutions (aq.) were prepared at concentrations of 0.1 and 0.3 M and sterile-filtered using a 0.22 μm PES filter prior to use for the final physical cross-link step. Briefly, each hydrogel sample was immersed in 3 mL of CaCl_2_ solution (aq.), allowing ionic gelation (10 min, 37 °C). CaCl_2_ solution (aq.) was removed, and samples were washed with HBS solution (3 times, 10 min, RT) to remove any excess of CaCl_2_. Prior to testing, hydrogels were equilibrated in F-12 medium at 37 °C in a humidified atmosphere with 5% CO_2_ for 24 h for complete swelling.^27^

#### 2.4.2 Rheological Tests

##### 2.4.2.1 Flow and Gelation Kinetics Tests

Rheological tests were first performed to measure flow properties and gelation kinetics of precursor hydrogel solutions (or biomaterial inks, Table 1) prior to gelation using a Thermo Scientific HAAKE MARS rheometer. Values were recorded by HAAKE RheoWin Job Manager (Version 4.87.0002). Flow properties were evaluated using a 20 mm cone plate geometry (C20/1° Ti L, 222–1877, Thermo Scientific), with a gap (plate-to-plate) adjusted to 0.1 mm, at a shear rate range 0.1–300 s^–1^. All tests were performed at 37 °C. A 35 mm parallel plate geometry was used to measure the gelation kinetics; the gap (plate-to-plate) was set to 0.1 mm prior to measurements. Oscillatory frequency and strain were kept constant at 1 Hz and 10%, respectively. The storage modulus (*G*′) and loss modulus (*G*″) were measured at 37 °C to measure the gelation kinetics and identify the gelation time of biomaterial inks.

##### 2.4.2.2 Oscillation Amplitude Tests

Rheological tests were performed to measure the viscoelastic properties of hydrogels (diameter 8 mm, height 5 mm, Table 1) by carrying out oscillation amplitude strain sweeps in the 0.01–100% strain range at a constant frequency of 1 Hz using a serrated 8 mm parallel plate geometry, at 37 °C, and with the gap (plate-to-plate) adjusted to the height of each sample. Retrieved data were analyzed using DIN 51810–2 recommended settings in RheoWin Data Manager (version 4.87.0002) to determine the values of *G*’ and *G*’’ in the linear viscoelastic range (LVR). All tests were performed using *n* = 3 samples for *N* = 3 independent experiments.

#### 2.4.3 Compression Tests

Compression tests were performed using a Texture Analyzer (Stable Micro Systems Texture Analyzer, TA-XT Plus) equipped with a 0.5 N load cell (Stable Micro Systems, Load: 538855) following our previous works.^27,31^ Cylindrical hydrogels were prepared (diameter 8 mm and height 5 mm) and equilibrated; prior to each test, the diameter of each sample was measured using a caliper to calculate the surface. Samples were compressed (0.1 mm/s) without precompression. The compressive moduli (i.e., Young’s modulus, E) was calculated from the stress–strain curve as the slope of the linear part of the curve and within a strain range of 0–5%. All tests were performed using *n* = 3 samples for *N* = 3 independent experiments.

#### 2.4.4 Hydrogel Porosity

The porosity of alginate-based hydrogels was investigated using the solvent replacement method.^24^ Cylindrical hydrogels were prepared, freeze-dried (0.01 mbar, −80 °C), and weighed (w_1_). Samples were immersed in an excess of absolute ethanol (24 h, RT) and weighed after excess ethanol on the gel was removed by blotting (w_2_). Porosity (%) was calculated using eq 1, in which ρ is the density of absolute ethanol and *V* corresponds to the volume of the sample. All tests were performed using *n* = 3 samples for *N* = 3 independent experiments.

1 $$\mathrm{Porosity}\left(\%\right)=\frac{w_{2}-w_{1}}{\rho V}\times 100$$

### 2.5 Prostate-Specific 3D In Vitro Models

#### 2.5.1 Cell Culture

PC-3 human prostate cancer cells were purchased from American Type Culture Collection (ATCC CRL-1435) and cultured in complete F-12 Ham cell culture medium (7% v/v FBS, 2 mM l-glutamine). PC-3 cells were discarded upon reaching passage 80. hTERT PF179T CAF human prostate cancer-associated fibroblast cells were purchased from American Type Culture Collection (ATCC CRL-3290TM) and maintained in complete EMEM (containing Earle’s Balanced Salt Solution and non-essential amino acids and supplemented with 10% v/v FBS, 2 mM l-glutamine, 1 mM sodium pyruvate, 1500 mg/L sodium bicarbonate 7.5%, and 10 mg/mL puromycin). hTERT PF179T CAFs (from now on referred to simply as CAFs) were discarded upon reaching passage 25. Coculture experiments were performed using complete F-12:EMEM medium (1:1 volume ratio) as reported in Supporting Information, SI.5. All cells were routinely cultured in an incubator (37 °C and 5% CO_2_).

#### 2.5.2 Preparation of Bioinks and Tissue-Specific 3D In Vitro Models

##### 2.5.2.1 Bioink Preparation

Sterile hydrogel precursor solutions **A** and **A-P** were used to prepare prostate and stromal bioinks by homogeneously mixing hydrogel precursor solutions in a 1:1 volume ratio with PC-3 (prostate bioinks) and hTERT PF179T CAF (stromal bioinks) at a concentration of 1 × 10^6^ cells/mL.

##### 2.5.2.2 Hydrogels and Tissue-Specific 3D In Vitro Models

Prostate and stroma bioinks were transferred in a 1 mL syringe and extruded through a 27G nozzle dropwise in the CaCl_2_ cross-linking solution, allowing gelation (10 min, RT), following previously reported procedures.^31^ After cross-linking, the formed hydrogel beads were collected using a cell strainer (CSS-010-040, Biofil, UK), transferred to HBS solution, and incubated (10 min, RT) to remove any excess of CaCl_2_. Finally, cell encapsulating hydrogel beads were transferred to complete cell culture media and cultured in an incubator (37 °C, 5% CO_2_).

#### 2.5.3 Bioprinting Engineered Prostate 3D In Vitro Models

Agarose fluid gels were used to support the printed construct during the printing process before cross-linking with CaCl_2_ solutions.^24,39^ Briefly, 0.5% w/v agarose fluid gels were prepared in dH_2_O by cooling autoclaved agarose from 100 to 25 °C under a constant shear of 700 rpm for at least 6 h. In a typical experiment, 2 mL/well of sterile fluid agarose gels were then loaded into 6-well tissue culture plates, and 3 mL of biomaterial ink was loaded into a printing cartridge using a piston to avoid undesired leakages and maintain sterility. The 3D Discovery Evolution bioprinter (RegenHU, Switzerland) was used to print engineered 3D prostate-specific in vitro models. Printing parameters were optimized using biomaterial inks **A** and **A-P**, as specified in Table 2. After printing, scaffolds were allowed thermal gelation and Schiff base reaction (10 min, RT), and then, the agarose gel was gently removed, leaving a very thin layer to prevent any possible damage. CaCl_2_ cross-linking solution (aq.) was added to the well, allowing the final ionic cross-linking step (10 min, 37 °C, 5% CO_2_). Finally, hydrogel scaffolds were gently washed with HBS (3 times, 10 min). For printing of engineered 3D prostate in vitro models, 3 mL of prostate and stromal bioinks were loaded into a printing cartridge, and the printing was performed with optimized printing parameters.

**Table 2:** Printing Parameters Used with a 3D Discovery Evolution Bioprinter to Optimize Manufacturing of Prostate-Specific 3D Models^a^

printing parameters	selected values
nozzle type	25G (steel, cylindrical)
printing speed (mm/s)	2.5, 5.0, 7.5, and 10.0
extrusion pressure (kPa)	20, 30, 40, 50, and 60

a The process was performed at a constant temperature (22 ± 3 °C).

### 2.6 Characterization of Engineered Prostate 3D In Vitro Models

#### 2.6.1 Cell Metabolic Activity

The Deep Blue Cell Viability Kit was used following the manufacturer’s instructions to determine the viability of encapsulated cells in the 3D in vitro models, which correlated with the measured metabolically active viable cells. Briefly and at each time point (i.e., 1, 4, and 7 days), one prostate 3D in vitro bead was placed in a 96-well plate, the cell culture media gently removed and replaced with 200 μL of 10% v/v deep blue viability solution in complete cell culture media, samples were incubated (2 h, 37 °C, 5% CO_2_), and then 100 μL of media were transferred to a new 96-well plate. Samples were read immediately using a plate reader spectrophotometer (BioTek, Synergy 2, NorthStar Scientific Ltd.) at an excitation range of 530–570 nm and an emission range of 590–620 nm. The measurements were performed using *n* = 5 samples for *N* = 3 independent experiments.

#### 2.6.2 Cell Viability

The Live/Dead assay was used following the manufacturer’s instructions to quantify the number of live and dead cells in all 3D in vitro models. At the selected time point, encapsulated cells in hydrogels were incubated (1 h, 37 °C, 5% CO_2_), washed twice with HBS, and fixed with 4% v/v PFA (30 min, RT), followed by final HBS washing. Images were acquired using a confocal fluorescence microscope using (Ex/Em 488/525 nm) and (Ex/Em 570/600 nm) filters to detect calcein (live cells, green) and ethylene homodimer (dead cells, red), respectively. Z-stack images were postprocessed using CQ1 software.

#### 2.6.3 Cell Marker Expression

To evaluate the role of the microenvironment on PC-3 cells, expression of selected markers (i.e., CD44, CD44v6, E-cadherin, and vimentin) was evaluated at different time points (i.e., 3 and 7 days). PC-3 cells cultured on standard tissue culture plates were used as a control (i.e., 2D controls). For the staining of 2D controls, the cells were washed with PBS and incubated with cell dissociation buffer (10 min, 37 °C, 5% CO2),^40^ whereas for 3D in vitro models, the cells were recovered by dissolution of hydrogels, as previously done by our group,^41^ and retrieved cell aggregates were disrupted with Accumax solution (10 min, RT). All cells were centrifuged at 600*g* (5 min, RT), gently resuspended in blocking buffer (5% (v/v) FBS in PBS), and incubated for 30 min on ice. For intracellular marker detection (i.e., vimentin), the cells were fixed with 4% PFA (10 min, RT) and washed three times with PBS, followed by a permeabilization step with 0.1% w/v Saponin in blocking buffer (30 min, RT). The cells were then incubated on ice with the primary antibody (45 min) and then with the secondary antibody (45 min) at concentrations reported in Table S2. Stained cells were analyzed using a flow cytometer (BD Fortessa X-20); data were analyzed performing a dead cell exclusion using DAPI staining (i.e., incubation of cells with 1 μg/mL DAPI in PBS), and positive cells were analyzed using FlowJo software (v10.8.0, BD). Single live cell gating was performed, obtaining the number of positive cells for each marker and calculating the median fluorescence intensity (MFI). Median intensity data are reported as an average of *N* = 3 independent biological experiments.

#### 2.6.4 Cell Phenotype and Aggregate Morphologies

The ability of PC-3 cells to form aggregates in different microenvironments was evaluated at selected time points (i.e., day 7 and day 14). Briefly PC-3 cells in prostate 3D in vitro models were fixed with a 4% PFA solution (aq.) (15 min, RT), washed with 1× HBS (*n* = 3), permeabilized with a 0.1% Triton X solution in HBS (15 min, RT), washed with PBS (*n* = 3), and incubated with a DAPI (1 μg/mL) and phalloidin Alexa Fluor 488 (1:80 v/v dilution in HBS) solution (45 min, RT). Finally, the samples were washed thrice with HBS and stored in HBS with antimycotic-antibiotic for image acquisition using (Ex/Em 405/447 nm) and (Ex/Em 488/525 nm) filters to detect DAPI (nuclei, blue) and phalloidin 488 (F-actin, green), respectively. Aggregates were identified by thresholding and converting images to a binary format and then selected using the ″create selection” function. Finally, the area and shape characteristics of the selected aggregates were measured using the “Analyze Particles” plug-in in ImageJ (v1.53a). Circularity was used as a shape descriptor to assess the resemblance of each object to a perfect circle, with a value of 1.0 indicating a perfect circle and a value of 0.0 indicating a highly elongated shape.

#### 2.6.5 Cell Adhesion

The ability of PC-3 cells to adhere to different ECM-mimicking materials after conditioning in different microenvironments (7 days) was evaluated. Briefly, collagen and fibronectin were used to coat 8-well chamber slides (80826, Ibidi) according to the manufacturer’s protocol. Collagen type I was diluted in a 17.5 mM acetic acid solution (aq) to 35 μg/mL. 200 μL was added to each well and incubated (1 h, RT), allowing absorption. Similarly, 200 μL/well of a 20 μg/mL fibronectin solution (aq) was used and incubated (1 h, RT). After this incubation step, solutions were removed, and wells were washed with sterile PBS and left to air-dry in sterile conditions (1 h, RT). PC-3 cells preconditioned in different hydrogels (Table 1) were recovered,^41^ seeded on uncoated (control) and collagen- or fibronectin-coated surfaces at a density of 1 × 10^4^ cells/cm^2^, and allowed to adhere to surfaces (1 h, 37 °C, 5% CO_2_). The cells were washed with PBS to remove non-adhered cells (*n* = 2), fixed with 4% PFA (15 min, RT), washed with PBS (*n* = 3), and further permeabilized with 0.1% Triton X in PBS (15 min, RT). The cells were incubated with 200 μL/well of 1 μg/mL DAPI in PBS and phalloidin (1:80 ratio of dilution) (45 min, RT), washed with PBS (*n* = 3), and stored in HBS with antimycotic-antibiotic for image acquisition using (Ex/Em 405/447 nm) and (Ex/Em 488/525 nm) filters to detect DAPI (nuclei, blue) and phalloidin 488 (F-actin, green), respectively.

### 2.7 Image Acquisition

#### 2.7.1 Brightfield

Brightfield images of encapsulated cells in hydrogel beads were acquired using a fluorescent inverted microscope (Leica DMI6000, Leica Microsystems, UK), connected to a 5.5 Neo sCMOS camera (Andor, UK). For image capturing, μManager software (v.1.46, Vale Lab, UCSF) was used. For acquisitions, a dry 2× objective (PLAN 2.5/0.07, Leica), a dry 10× objective (PL 10/0.3 PH1, Leica), and a dry 20× objective (PL 20/0.5 PH2, Leica) were used.

#### 2.7.2 Laser Scanning Confocal

Images were acquired using a confocal microscope (CQ1 Confocal Imaging Cytometer Yokogawa) coupled with a sCMOS camera (2000 × 2000 pixels, 13.0 × 13.0 mm) and using microlens-enhanced wide-view (Nipkow disk). For acquisitions, 4×, 10×, and 20× objectives and (Ex/Em 405/447 nm), (Ex/Em 488/525 nm), and (Ex/Em 570/600 nm) filters were used. For all samples, Z-stacks were acquired with a 5 μm z-step.

### 2.8 Statistical Analysis

All results were reported as mean ± SD. For statistical analysis, all results were analyzed with two-way ANOVA using GraphPad Prism v9.5.1. *P*-values were set at four different significance levels: *p ≤ 0.05, **p ≤ 0.01, ***p ≤ 0.001, and ****p ≤ 0.0001.

## 3 Results and Discussion

### 3.1 Biomaterial Ink and Prostate-Specific Hydrogel Characterization

#### 3.1.1 Oxidized Alginate

Oxidation of alginate was confirmed by ^1^H NMR spectra, showing the typical alginate fingerprint with peaks in the region from 3.6 to 4.0 ppm, corresponding to the protons of both G and M units (Figure 1). As the oxidation involves only the G units, the OA_50_ spectrum shows a signal at 4.2 ppm with distinctive peaks at 5.35 and 5.60 ppm (Figure 1), both attributed to the hemiacetalic proton formed after oxidation.^42^ Aldehyde signals expected at 9.7 ppm^43^ were not detected, possibly due to the hemiacetal formation of aldehyde groups with the adjacent hydroxyl groups, as explained previously.^26^ The degree of oxidation achieved (target value DO = 50%) was quantified using the triiodide-starch method, and an aldehyde concentration of approximately 6.5 μM/mg was detected, confirming values reported in our previous study.^26^

**Figure 1:**
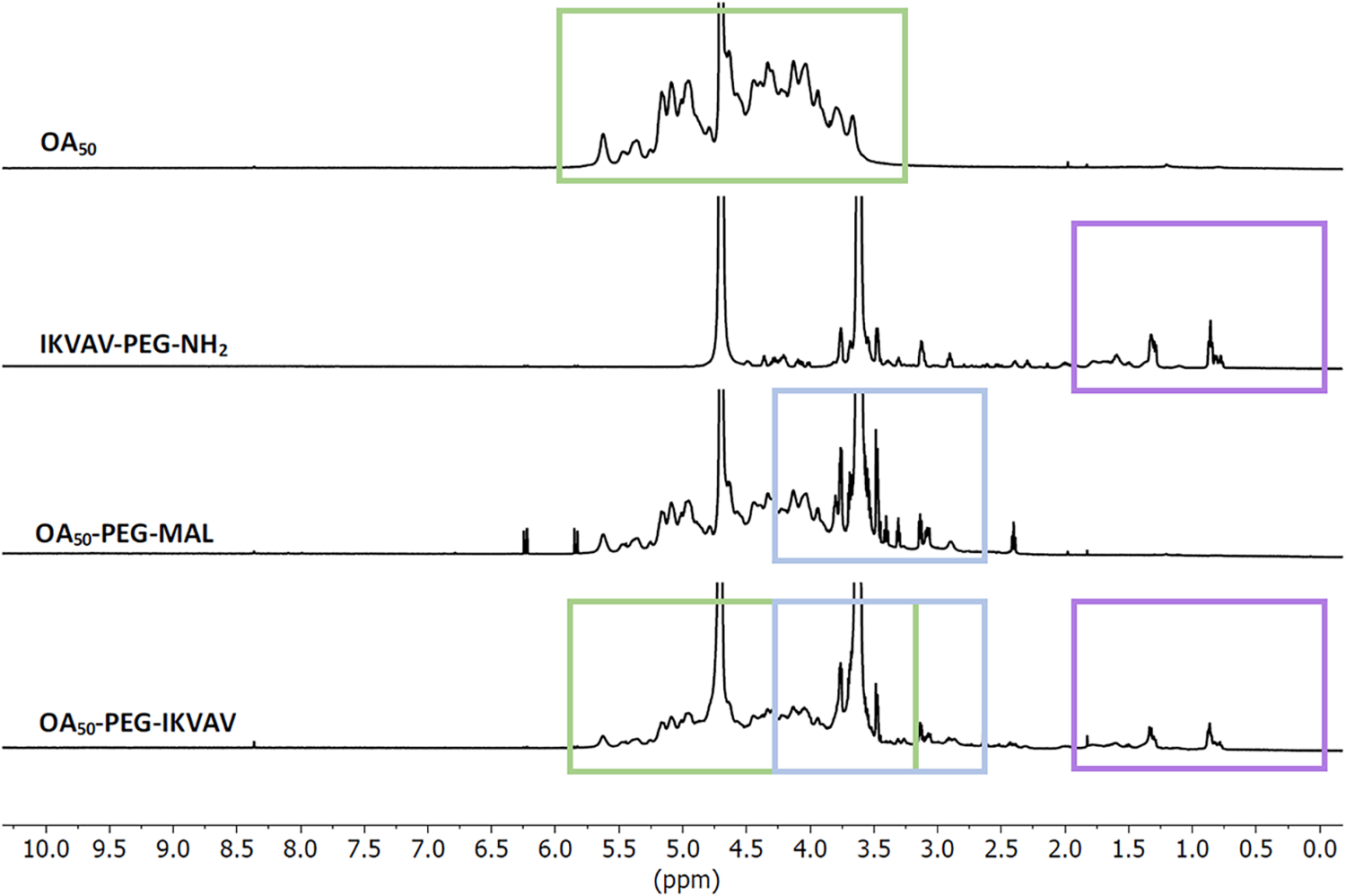
Characterization of functionalized alginate. ^1^H NMR spectra of oxidized alginate OA_50_, IKVAV-PEG-NH_2_, OA_50_-PEG-MAL, and OA_50_-PEG-IKVAV.

#### 3.1.2 Functionalized Alginate

The functionalization of OA_50_ was characterized using a selected laminin-like peptide and assessed via H^1^ NMR spectra to confirm the synthesis of OA_50_-PEG-IKVAV (Figure 1). Prior to alginate functionalization, MAL-PEG-NH_2_ and the selected thiol-terminated peptide (IKVAV-SH) were characterized by H^1^ NMR, with the spectra showing that MAL-PEG-NH_2_ has characteristic peaks at 3.0–4.0 ppm, attributed to PEG, and around 6.70 ppm, representing the maleimide group,^44^ with 5.98 and 6.38 ppm, attributed to the double bond protons of the maleimide group.^45^ The IKVAV-SH spectrum reports characteristic peaks of valine, isoleucine, and alanine in the range of 1.5–0.5 ppm^46^ (Figure S1A). The conjugation between MAL-PEG-NH_2_ and OA_50_ was then optimized using two concentrations of MAL-PEG-NH_2_ (100 and 200 μM; Figure S1B) by comparing the ratio of the integrals for H^1^ NMR reference (TMSP) protons to the methyl protons from PEG’s terminal methoxy group (δ ∼ 3.2 ppm): a coupling efficiency of approximately 70% was obtained for both concentrations. Results confirmed that 100 μM MAL-PEG-NH_2_ is sufficient to achieve conjugation with OA_50_ (4% w/v), confirming what was reported in the literature.^38^

Then, the coupling of MAL-PEG-NH_2_ and the selected thiol-terminated peptide (IKVAV-SH) was quantified by Ellman’s assay, using cystine for the standard calibration curve. This assay was used to monitor the reaction kinetics and sample reaction products after 1.5 and 2.5 h. An increase was observed in the coupling efficiency from 80% (reaction time of 1.5 h) to 99% after 2.5 h of reaction (data not shown). Based on these results, it was decided to incubate the thiol-terminated peptide with MAL-PEG-NH_2_ for 3 h to complete the conjugation via a Michael-type addition reaction and ensure complete coupling between the reagents.

Finally, H^1^ NMR was used to confirm the functionalization of alginate with laminin-mimicking thiol-terminated peptides. As shown in Figure 1, the OA_50_ spectrum reports the typical peaks of OA in accordance with our previous study,^26^ whereas the IKVAV-PEG-NH_2_ intermediate product shows clearly all of the peaks corresponding to both IKVAV and PEG, confirming the coupling. Further, the reduced signal assigned to the maleimide group (at δ 5.9–6.5 ppm) indicates that a reaction with thiols occurs (Michael-type addition reaction), confirming the coupling of IKVAV-PEG-NH_2_ with OA_50_: the region >2.0 ppm in the OA_50_-PEG-IKVAV matches the IKVAV signal, which is not displayed in the corresponding OA_50_-PEG-Mal signal (used as control).

### 3.2 Physical Characterization of Prostate-Specific Alginate-Based Hydrogels

#### 3.2.1 Porosity and Pore Size

The porosity of hydrogels is a physical property that influences prompt water uptake and cell migration.^47^ In this study, hydrogel porosity was evaluated by solvent replacement (Figure 2A), and pore size was quantified by SEM micrographs (Figure S3). Hydrogels **A1** and **A1-P** have higher porosity (approximately 10%), reflecting the lowest cross-linking density linked to the lowest CaCl_2_ (aq.) concentration, whereas hydrogels **A3** and **A3-P** have lower porosity (Figure 2A). As expected, it is possible to evidence that a lower cross-linker concentration (100 μM CaCl_2_) caused the highest porosity, whereas hydrogel **A3** is the sample with the lowest porosity, as the hydrogel formulation has a higher concentration of the ionic cross-linker (300 μM CaCl_2_) and the presence of additional cross-links between gelatin and OA_50_ (Supporting Information 2, Figure S2). Additionally, hydrogels **A1** and **A1-P** showed a bigger pore diameter size compared to **A3** and **A3-P**, respectively (Figure S3). This was in accordance with the porosity results. All of the hydrogels showed interconnected porosity with values of the pore diameter in the range of 50–800 μm (Supporting Information 4).

#### 3.2.2 Mechanical Properties of Prostate-Specific Hydrogels

Tissue stiffening in PCa is attributed to variations in ECM composition due to increased collagen deposition, being responsible for controlling mechano-signaling to cancer cells during tumor progression.^6,35^ Based on values reported in the literature on the mechanical properties of human prostate tissues,^33–37,48^ we formulated hydrogels addressing both healthy (E ∼ 2 kPa) and advanced/metastatic PCa (E > 10 kPa).

Compression tests were performed with no initial prestress to quantify Young’s modulus (E), with measured values in the range of 2.5–13.0 kPa (Figure 2B). As expected, the results show that E is proportional to CaCl_2_ (aq.) concentration and hence cross-linking density. Moreover, the presence of PEG on alginate chains causes a decrease in measured E, possibly due to the increased water content in hydrogels and overall loosening of the hydrogel network. As anticipated, hydrogels **A1** and **A3** have increased cross-linked densities due to the covalent cross-links OA_50_ and gelatin (Schiff base formation between amino groups and aldehyde groups, Figure S2) that increase E compared to hydrogels **A1-P** and **A3-P**. In fact, hydrogel **A1** is stiffer than hydrogel **A1-P** (5.6 ± 0.6 kPa vs 3.0 ± 0.5 kPa, *P* ≤ 0.0001) even if cross-linked with the same CaCl_2_ (aq.) concentration (100 μM); a similar trend is observed between hydrogels **A3** and **A3-P** (12.6 ± 1.3 kPa vs 6.6 ± 0.7 kPa, *P* ≤ 0.0001) when using a higher CaCl_2_ (aq.) concentration (300 μM).

**Figure 2:**
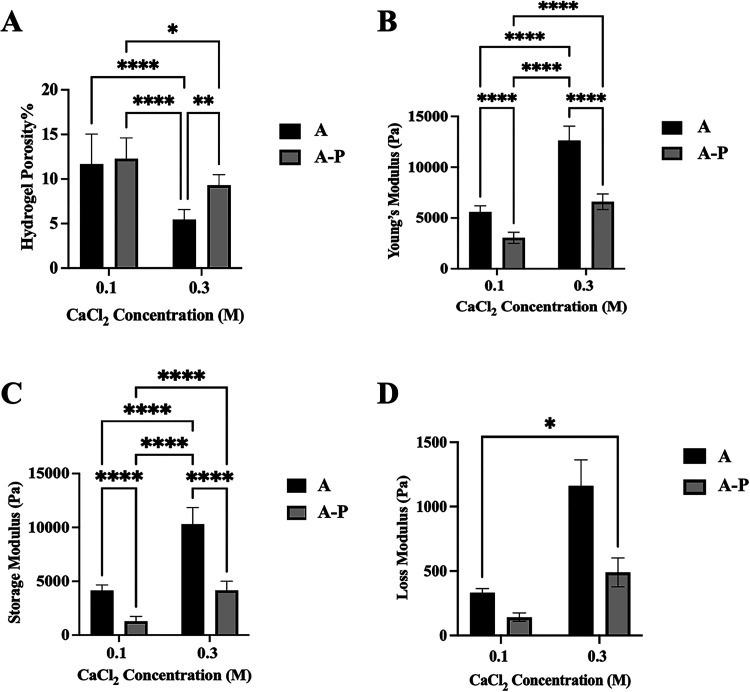
Physical and mechanical properties of alginate-based hydrogels. (A) Porosity percentage of alginate-based hydrogels. (B) Young’s modulus measured by uniaxial compressive tests. All hydrogels and CaCl_2_ concentration are significantly different (*p* ≤ 0.0001). (C) Storage and (D) loss modulus measured with a rheometer at a constant frequency (1 Hz). All hydrogels and CaCl_2_ concentrations are significantly different (*p* ≤ 0.0001). Data presented as mean ± SD (*N* = 3, *n* = 3) for all samples. *P*-values represented as *p ≤ 0.05, **p ≤ 0.01, ***p ≤ 0.001, and ****p ≤ 0.0001.

Rheological test results align with the trend shown in compression tests, with measured storage moduli (*G*′, Figure 2C) being higher in hydrogels **A1** and **A3** (4.1 ± 0.5 kPa, 10.3 ± 1.5 kPa) than in hydrogels **A1-P** and **A3-P** (1.3 ± 0.4 kPa, 4.2 ± 0.8 kPa), with increased *G*’ at higher CaCl_2_ (aq.) concentrations (*P* ≤ 0.0001) and reduced *G*’ in the presence of PEG in alginate chains (*P* ≤ 0.0001). The loss modulus (*G*”, Figure 2D) measured in shear sweep tests follows the same trend as *G*’, with the highest value in hydrogel **A3** (1.2 ± 0.2 kPa) and the lowest in hydrogel **A1-P** (0.1 ± 0.0 kPa). CaCl_2_ (aq) concentration is again proportional to the loss modulus: hydrogels **A1** and **A3** (0.3 ± 0.0 kPa vs 1 ± 0.2 kPa, *P* ≤ 0.0001) have higher loss moduli than hydrogel **A1-P** and **A3-P** (0.1 ± 0.0 kPa vs 0.5 ± 0.1 kPa, *P* ≤ 0.0001).

Of note and as reported, the addition of PEG to alginate chains causes higher loss moduli of the hydrogels, which is reported to be proportional to PEG concentration and molecular weight,^49^ which was not investigated in this study.

### 3.3 Manufacturing of Prostate-Specific 3D In Vitro Models

#### 3.3.1 Biomaterial Inks: Rheological Properties, Printability, and Gelation Kinetics

When it comes to 3D printing, biomaterial inks should meet specific requirements in the flow properties (i.e., shear thinning behavior), allowing extrudability and shape retention for ease of manufacturing of 3D models with bioprinters.^50^ Here, the flow properties of prostate biomaterial inks were evaluated with a rheological test, measuring the dynamic viscosity in response to the shear rate and assessing their shear thinning properties.^51^ As reported in Table 1, two biomaterial inks (or hydrogel precursor solutions) were tested, both showing shear thinning behavior (Figure 3A,B). As per the hydrogel formulation design, biomaterial inks have two components that react to form covalent cross-links (Schiff base reaction between OA_50_ and gelatin), allowing hydrogel formation: this reaction causes a variation in biomaterial ink viscosity over time. Therefore, gelation kinetics was monitored through rheological tests to identify the printability window, in which no effect of the Schiff base reaction is observed. The gelation kinetics of prostate biomaterial inks show that the *G*’ value of biomaterial ink **A** is higher than that of biomaterial ink **A-P** already after mixing (0.25 ± 0.16 and 0.07 ± 0.03, respectively). Rheological tests show an average gelation time of biomaterial ink **A** of 24.5 ± 3.5 min at the crossing point (i.e., *G*’ = *G*”: 5.9 ± 0.9 Pa; Figure 3C,D). Biomaterial ink **A-P** has a gelation time of 43.4 ± 2.1 min at the crossing point (i.e., *G*’ = *G*”: 4.7 ± 0.8 Pa, Figure 3C,D). Such a difference in gelation time and cross-linking point value (5.9 ± 0.9 Pa vs 4.7 ± 0.8 Pa) confirms the lower availability of aldehyde groups to react with primary amines of gelatin. As a result, to ensure constant dynamic viscosity values during printing, biomaterial inks are prepared and used within 10 min after preparation.

**Figure 3:**
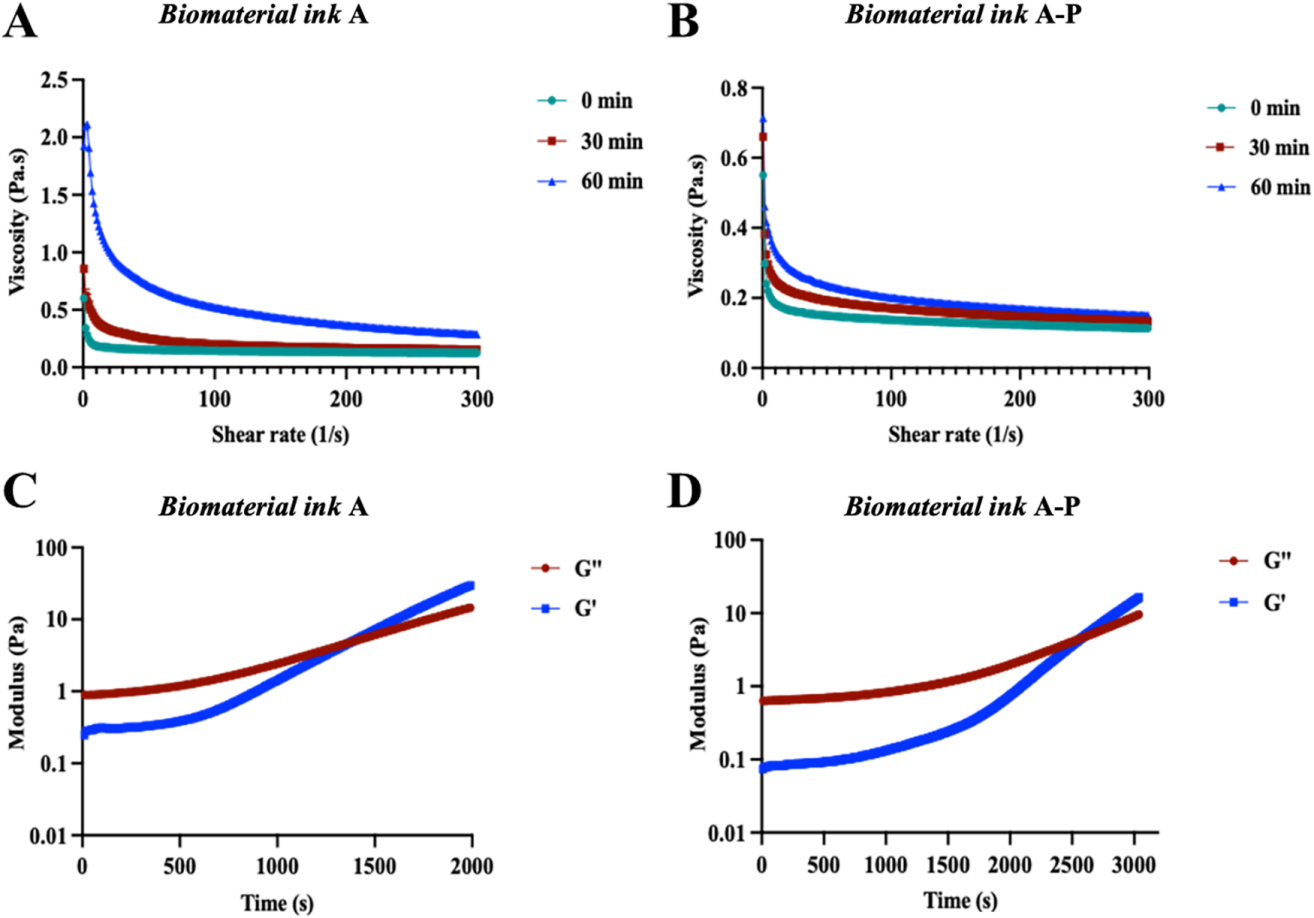
Flow curve and gelation kinetics. (A, B) Viscosity as a function of shear rate (37 °C) for biomaterial inks **A** and **A-P**, respectively. (C, D) Time sweep rheology of biomaterial inks. Over time, the storage modulus (*G*′) and loss modulus (*G*″) were measured at 37 °C to obtain gelation time post mixing the gel components, where gelation time was recorded at the crossover of *G*′ and *G*′′. Data are presented as the mean of *N* = 3, *n* = 3.

#### 3.3.2 Prostate-Specific 3D In Vitro Models

The cell viability and reorganization of PC-3 cells and CAFs was evaluated in prostate-specific alginate hydrogel microenvironments, assessing any possible influence of biomechanical and biochemical elements of the TME. First, an optimization step of the cell culture media was performed to ensure effective coculture of PC-3 cells and CAFs (Figure S4). Representative brightfield images of prostate-specific microenvironments as hydrogel **A3-P** are shown at different time points (i.e., days 1, 4, and 7) in Figure 4A. Regardless of the bioink used (either prostate or stroma bioink), prostate-specific microenvironments are stable over time (Figure S5), with the viability of PC-3 cells confirmed by the Live/Dead assay (Figure S6). To confirm this observation, it is observed that PC-3 metabolic activity increases over time (days 1, 4, and 7, Figure 6B), with a higher proliferation rate of PC-3 cells in hydrogel **A1-P**.

**Figure 4:**
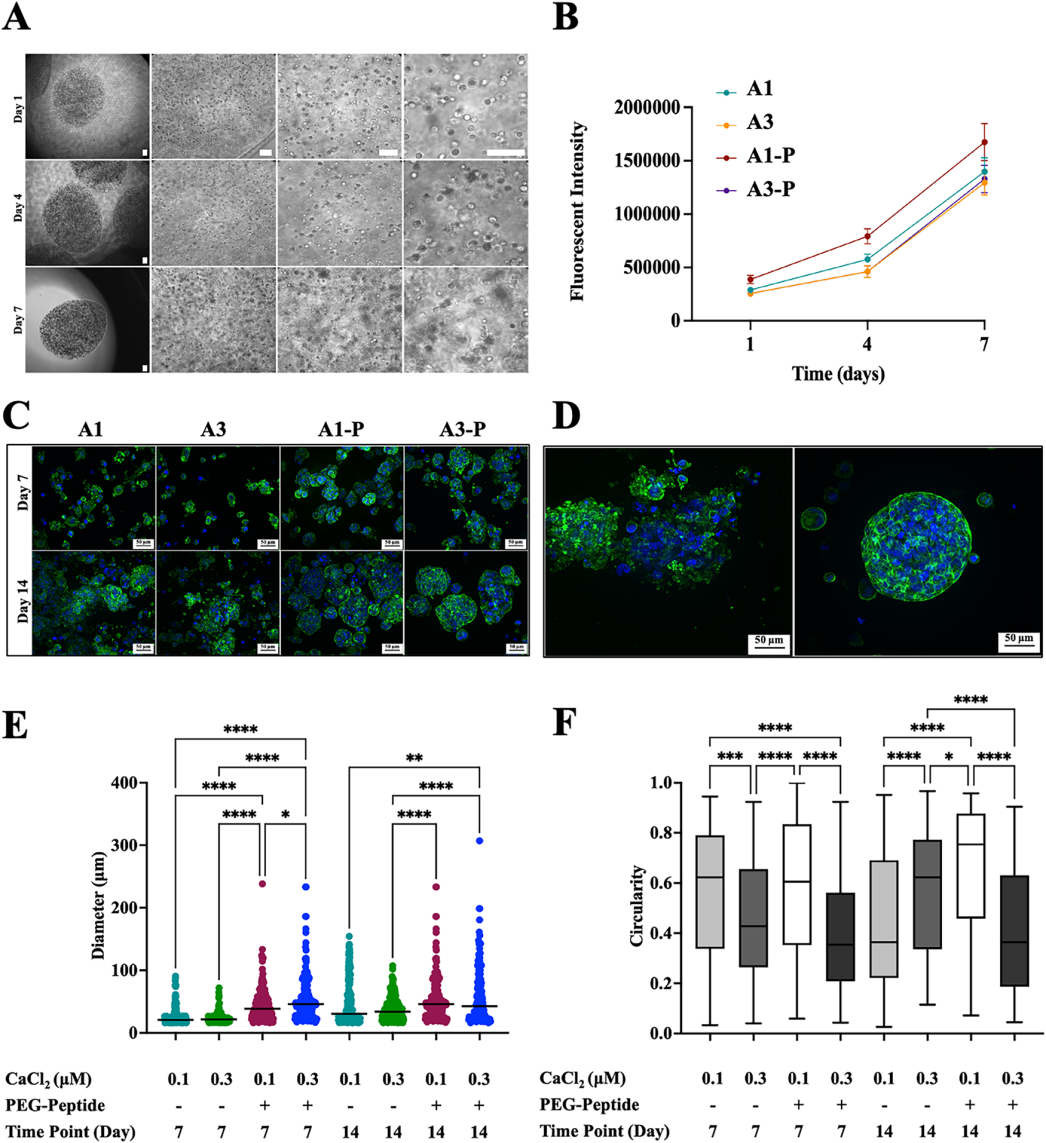
PC-3 prostate 3D in vitro models. (A) Representative images of PC-3 cells encapsulated in hydrogel **A3-P**. Images acquired with 2×, 10×, 20×, and 40× objectives, respectively, at days 1, 4, and 7. Scale bars: 100 μm. (B) PC-3 cell viability at different time points (days 1, 4, and 7). (C) Immunofluorescent images of PC-3 cells stained with DAPI (nuclei, blue) and phalloidin (F-actin, green) showing cell aggregates. (D) Representative images of PC-3 cell aggregates with low (left, **A3** hydrogel) and high (right, **A1-P** hydrogel) circularity values at day 14. Scale bars: 50 μm. (E) Quantification of the diameter (μm) of aggregates and (F) of the circularity of aggregates. Values are represented as mean and SD, with at least *n* = 200 aggregates for each tested condition. Data are presented as mean ± SD (*N* = 3, *n* = 3), *P*-values represented as *p ≤ 0.05, **p ≤ 0.01, ***p ≤ 0.001, and ****p ≤ 0.0001.

Alginate-based hydrogels were herein engineered to offer a versatile platform that provides control over mechanical properties and chemical composition (Table 1 and Figure 2) and as a tool to obtain in vitro tissue models for the exploration of fundamental mechanisms in health and disease. When cultured in distinctive microenvironments, PC-3 cells aggregate differently when cultures in prostate-specific microbeads varying in composition and mechanical properties are shown in Figure 4C, as previously observed in our previous studies in breast-specific microenvironments;^31^ cell aggregates vary in size and shape over time (day 7 and day 14), showing an in vivo-like tumor structure as a response and adaptation to the microenvironment.^52^ As shown in Figure 4C, PC-3 cells started to form cellular aggregates at day 7 in all hydrogels with pronounced formation in hydrogels **A1-P** and **A3-P** compared to hydrogels **A1** and **A3** (*p* ≤ 0.0001). In the latter hydrogels, few cell aggregates are observed, with a majority of cells remaining dispersed in the hydrogel or forming smaller aggregates. In all hydrogels, cells form larger aggregates over time (day 7 vs day 14), suggesting sustained cell proliferation. The shape of aggregates is scored as circularity, for which 1 is assigned to objects perfectly fitting a circular structure and 0 to irregular ones (Figure 4F). PC-3 cells form grape-like structures with high circularity in hydrogel **A1-P** (*p* ≤ 0.0001 vs hydrogel **A3-P**), as also observed in other works using chitosan–chondroitin sulfate and Matrigel scaffolds.^53,54^ A reduced circularity is observed at day 14 in both hydrogels **A1-P** and **A3-P** compared to day 7 (Figure 4D,E), which suggests a morphological transformation of round aggregates into more invasive structures at day 14 and the observation of bridge-like structures connecting cell aggregates.^54^

#### 3.3.3 Bioprinting Engineered 3D Prostate In Vitro Models

Maintaining a good shape fidelity and shape retention while using low-viscosity biomaterial inks is challenging: to tackle this problem, suspension bath-based bioprinting was used to print low-viscosity biomaterial inks.^55^ Briefly, suspension bath-based bioprinting relies on a fluid-like gel with homogeneous particle distribution, shear-thinning, and self-healing properties to enable the movement of the printing needle without disrupting the printed construct until cross-linked. The printing parameters were optimized for biomaterial ink **A** and biomaterial ink **A-P**, without the inclusion of cells (bioinks). Biomaterial inks were printed under different conditions (Table 2), and the filament diameter was calculated from images (Figure 5A,B). A continuous filament of biomaterial ink **A** is obtained with low extrusion pressure (20 to 30 kPa) and a high feed rate (7.5 to 10 mm/s) (Figure 5B). As expected, for both biomaterial inks, at all extrusion pressures, the increased feed rate led to a decrease in filament diameter, reaching dimensions comparable to the nozzle diameters in few conditions (Figure 5C,D), as also reported elsewhere.^50^ hTERT PF179T CAFs were cocultured with PC-3 cells to better recapitulate the prostate TME and evaluate the role of stromal cells in PCa progression. To assess the feasibility in controlling the spatial location of PCa cells (i.e., PC-3) and CAFs (i.e., hTERT PF179T CAFs), prostate bioinks and stromal bioinks were printed using optimal printing parameters selected as 20 kPa extrusion pressure and 7.5 mm/s feed rate. Cell viability assessed using Live/Dead staining 1 day and 7 days after printing confirms that the cell viability of PC-3 cells is higher than 90% (Figure 5E,F) and confirms the possibility of using bioprinting to further engineer 3D prostate in vitro models.

**Figure 5:**
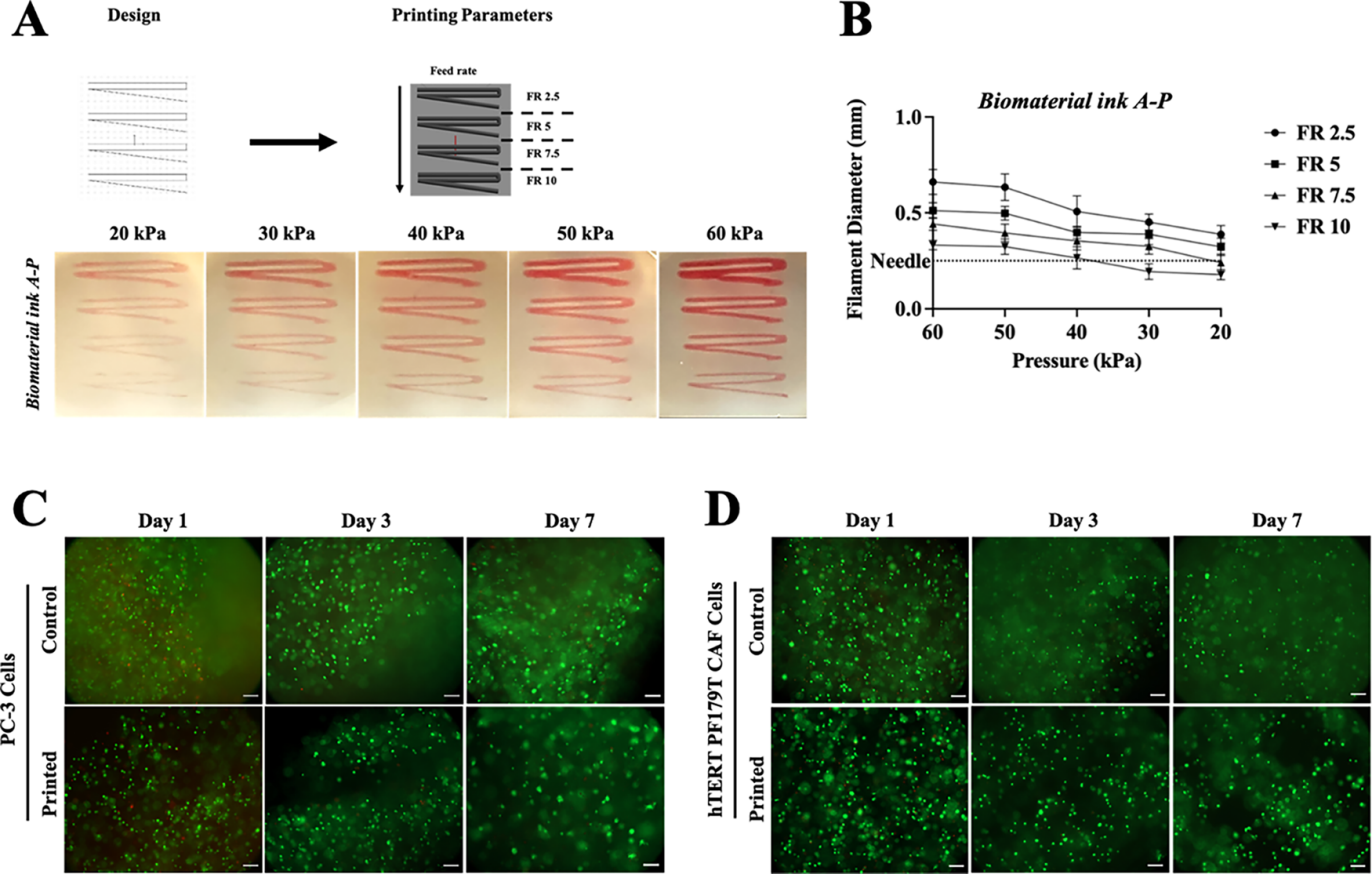
3D printed prostate-specific in vitro models. (A) 3D bioprinting parameters optimization via extrusion-based bioprinting. (B) Quantification of filament diameter. Data are plotted as mean ± SD (*N* = 2, *n* = 3). (C, D) Live/dead assay of printed cells: printed PC-3 (C) and CAF cells (D) at days 1, 3, and 7 were compared to control encapsulated cells to evaluate the effect of shear stresses during the printing process. In the image, live proliferative cells are displayed in green, whereas dead cells are displayed in red. Scale bars: 200 μm.

### 3.4 Effect of Prostate-Specific TME on PC-3 Prostate Cancer Cell Phenotypes

New approach methodologies (NAMs) are herein used to bioprint 3D engineered prostate-specific in vitro models, with a high impact for further use in assessing the possibility of linking mechanistic investigations in vitro with clinical data and possibly predicting clinical outcomes. PC-3 cells showed different behaviors when cultured in different prostate-specific microenvironments (Figure 4), forming larger and more circular cell aggregates when cultured in prostate-specific hydrogels (i.e., **A1-P** and **A3-P**). Engineered PCa TMEs were obtained using suspension bath-based bioprinting and controlling the architecture, the biochemical composition, and mechanical properties of prostate ECM, as well as the localization and density of PCa and stromal cells (i.e., PC-3 and CAFs were printed with a 1:1 cell number ratio). The impact of PCa cells and the prostate ECM on metastatic potential was evaluated on PC-3 cell behaviors (e.g., adhesion, migration, invasiveness) and phenotypes at different time points, allowing PC-3 cells to adapt to the engineered TME.

#### 3.4.1 Adhesion on Different ECM-Mimicking Interfaces

The first key process in PC-3 adaptation to different prostate-mimicking ECM (hydrogels in Table 1) and the presence of stromal cells (i.e., monoculture, coculture with CAFs) evaluated was PC-3 cells’ ability to adhere to different surfaces coated with ECM-relevant components, namely, collagen and fibronectin, compared to uncoated surfaces. Quantitative evaluation of PC-3 cell adhesion was performed by comparing the number of adhered cells and their spreading area among selected surfaces and, after 1 week, PC-3 cell adaptation to the engineered TMEs. These 2D tests were designed to validate cellular adhesion, which normally anticipates migratory and invasive behavior.

PC-3 cells were allowed to adhere for 1 h to different surfaces (i.e., tissue culture-treated, collagen, fibronectin) after preconditioning in different engineered microenvironments and then stained to evaluate their morphology (Figure 6A). Among all of the engineered prostate 3D in vitro models, the highest number of adhered PC-3 cells is recorded after preconditioning in hydrogel **A3-P** (E = 6.6 ± 0.73 kPa), where the presence of CAFs in all surfaces tested did not significantly impact the number of adhered PC-3 cells (Figure 6B) and with the highest number of adhered cells identified on collagen-coated surfaces (Figure 6C). Of note, conditioning TME does not highly impact PC-3 cells adhered to uncoated wells, whereas a certain level of correlation between conditioning TME and adhesion surface is observed with fibronectin- and collagen-coated surfaces (hydrogel **A3-P** > **A3** ≥ **A1-P** > **A1**); similar trends are observed in both coated surfaces in the presence of CAFs. Overall, it is not just the biomechanical properties (e.g., stiffer ECM) that drive cell adhesion capacity, but rather the biochemical elements (i.e., presence of α-laminin peptides) in which PC-3 cells are preconditioned. These results align with formation of PC-3 cell aggregates (Figure 4C) with less circular and more invasive morphologies in hydrogels **A3** and **A3-P**, suggesting that a stiffer ECM promotes expression of an invasive cell phenotype. The cell spread area (Figure 6D) further confirms that both hydrogel stiffness (E ≤ 6 kPa) and composition (presence of α-laminin peptides) increase the migration and invasion potential of PC-3 cells. Of note, formation of PC-3 cell protrusions is observed, indicative of preliminary stages of migration and invasion (data not shown).^48,56^

**Figure 6:**
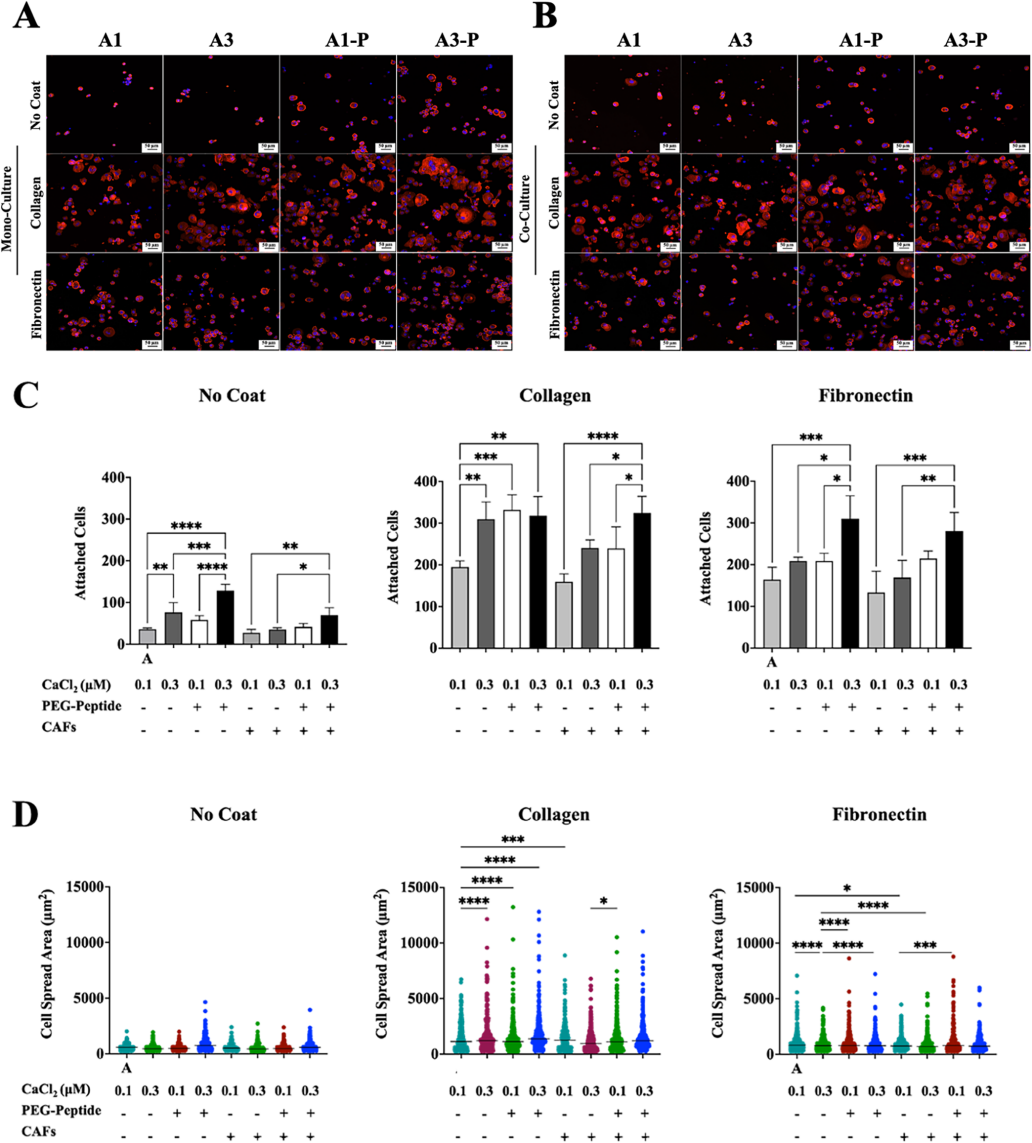
PC-3 cell adhesion on different substrates without/with CAFs. (A, B) Immunofluorescent images of PC-3 cells preconditioned in hydrogels without/with coculture with CAF cells. Cells are stained with DAPI (nuclei, blue) and phalloidin (F-actin, red), then allowed to adhere to different surfaces, noncoated, collagen-coated, or fibronectin-coated surfaces (scale bars: 50 μm). (C) Count of attached cells to different surfaces in different models. (D) Dot plot representations of individual cell spread area and mean in different surfaces when cells are allowed to adhere for 1 h. The labels in graphs are denoted A (hydrogel **A1**), B (hydrogel **A3**), C (hydrogel C1), and D (hydrogel C3). *P*-values represented as *p ≤ 0.05, **p ≤ 0.01, ***p ≤ 0.001, and ****p ≤ 0.0001.

#### 3.4.2 Epithelial-To-Mesenchymal Transition (EMT) Markers and CD44 Expression

Examining phenotypic changes in PCa cells is essential to understand aggressiveness and metastatic potential; with epithelial-to-mesenchymal transition (EMT) being recognized as a pivotal mechanism in PCa progression and predictive in determining clinical outcomes. Among the EMT markers used to evaluate PCa progression in PC-3 cells, we selected to evaluate the expression of E-cadherin, vimentin, CD44s, and CD44v6.^57,58^

Clinical evidence suggests a correlation between specific TME traits and PCa aggressiveness, with a shared underlying thread being that the higher the Gleason score, the more aggressive the cancer. In this, ECM interaction with PCa cells drives their phenotypic variations over time, as well as the presence of stromal cells.^59,60^ As expected, expression of markers in PC-3 cells cultured in 3D in vitro models was different from when cultured in 2D (Figure S7). Remarkably, PC-3 cells in 3D in vitro models have higher expression of vimentin, with CD44 expression being positively correlated to hydrogel stiffness. In 3D in vitro models, we also found that PC-3 cells express increased levels of vimentin when cocultured with CAFs, with a limited impact of the ECM traits (Figure 7A). Of note, PC-3 cells are found positive for vimentin (>80%) in all tested 3D in vitro models and time points, in line with what was reported by Xu et al.^61^ Of note, PCa cells positive for CD44 express a higher level of vimentin;^57^ when PCa cells interact with CAFs, they tend to express higher levels of vimentin: this corroborates with expression of the CD44 of PC-3 in this study (Figure 7C). We observed, as reported in the literature, that the EMT process in PC-3 cells is also linked to the loss of CD44v isoforms, which coincide with reduced E-cadherin (Figure 7B) and increased mesenchymal markers like vimentin.^57^ In all tested conditions, E-cadherin MFI is found to be constant in PC-3 cells, with a higher number of positive cells found in the presence of CAFs (approximately 40% vs approximately 50%).^62^

**Figure 7:**
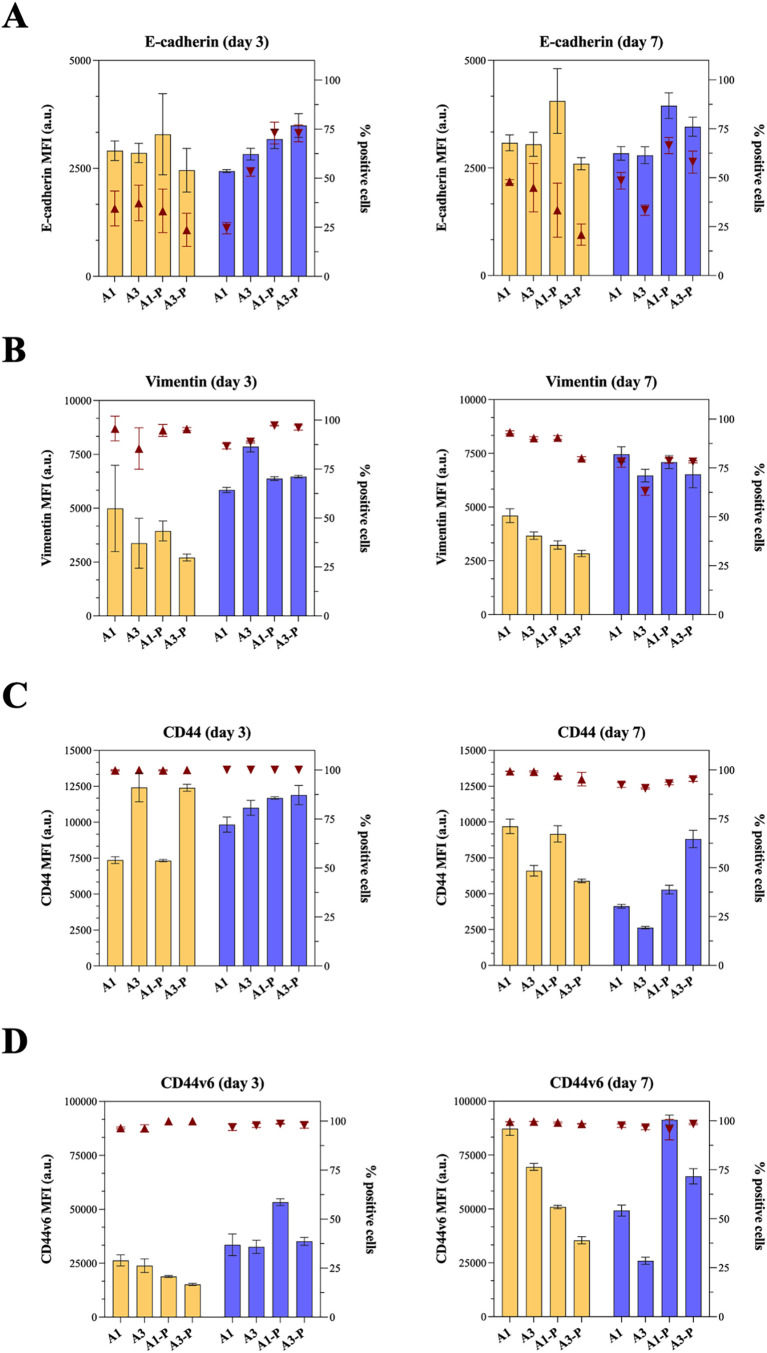
Flow cytometry analysis of EMT markers and CD44 in PC-3 cells cultured in a 3D in vitro model showing median fluorescence intensity (MFI, left *y*-axis) and the percentage of positive cells (right *y*-axis, represented as dark red triangles) of (A) vimentin, (B) E-cadherin, (C) CD44, and (D) CD44v6. Values are represented as mean and SD of *N* = 3 independent experiments.

Dysregulated CD44 expression characterizes most human cancers, including PCa. We observed an increase in CD44 expression in stiffer hydrogel **A3** (*p* ≤ 0.0001) and hydrogel **A3-P** (*p* ≤ 0.0001) with E values ranging from 12.6 to 6.6 kPa after 3 days in engineered TME when PC-3 is cultured alone, compared to softer hydrogels A1 and C1 with E values ranging from 5.6 to 3.0 kPa (Figure 7C). At the same time point, when PC-3 cells are cocultured with CAFS, this trend is not observed with high CD44 expression, with >95% found positive for the standard CD44 isoform. Interestingly, the trend is inverted after 7 days for PC-3 in a monoculture: in softer ECM (i.e., hydrogels **A1** and **A1-P**), CD44 expression increases and is higher than that in PC-3 cells cultured in stiffer ECM (i.e., hydrogels **A3** and **A3-P**). An overall reduction in CD44 expression in PC-3 cocultured with CAFs is observed, with no correlation with the biomechanical and biochemical traits of the hydrogels.

Clinically, CD44 expression tends to decrease as PCa progresses to higher Gleason grades and more poorly differentiated, aggressive tumors. Such an association suggests that with higher vimentin and lower E-cadherin expression (i.e., PC-3 EMT), reduced CD44 expression is associated with aggressive traits in PC-3 cells. Overall, in the presence of CAFs, CD44 decreases in PC-3 cells, showing a more invasive/aggressive phenotype after 7 days, suggesting a higher metastatic potential of PC-3 cells. Further investigation correlating CD44/CD24 and PC-3 cancer stemness may be of use to better interpret their invasiveness. On this note and based on our previous findings on breast cancer cell invasiveness,^31,32^ we investigated the expression of CD44v6 isoform typically implicated in tumor cell invasion and metastasis.^57^ Expression of CD44v6 in PC-3 cells (>95%, across all of the conditions tested) confirms the aberrant increase of CD44 variant isoforms with the loss of CD44s expression as PCa progresses. Markers of tumor differentiation and progression show that among the different TMEs tested, stiffer and laminin-enriched ECMs (i.e., hydrogel **A3-P**) are overall associated with the invasive traits of PC-3 cells, with and without CAFs. This aligns with adhesion results, in which stiffer and laminin-enriched ECM promotes higher PC-3 adhesion on the substrates (Figure 6). To follow up on this, we further investigated migration and invasiveness of PC-3 cells, observing similar behaviors: at day 3, PC-3 cells from hydrogel **A3-P** have significantly higher migration capability compared to other TMEs (Figures S8 and S9) and a possible influence of CAFs on the migratory ability of PC-3 cells. We assessed PC-3 cells’ ability to invade collagen ECM at early time points (Figure S9), but none of the conditions tested at this early time point showed a distinguished invasive trait of PC-3 cells. To better investigate the role of TME in cell invasive phenotypes, longer observational time points may be required.

## 4 Conclusions

This work shows the potential of engineered prostate 3D in vitro models as new technologies to decouple and analyze specific biomechanical and biochemical properties of the PCa TME. By leveraging functionalized alginates and bioprinting, reproducible, spatially defined constructs were manufactured as specific 3D in vitro PCa models, enabling the systematic investigation of how TME properties direct PCa cell aggressiveness and invasiveness.

Our approach successfully functionalized alginate with laminin-mimicking peptides (AG73, IKVAV) via PEG to create hydrogels with tunable viscoelastic properties (2.5–13 kPa) that match the stiffness of both healthy and tumoral prostate tissues. Biological validation confirmed that 3D in vitro models support the viability and proliferation of cells and enable quantification of the expression of tumor markers (i.e., CD44, CD44v6, vimentin, and E-cadherin).

Notably, hydrogels enriched with laminin peptides significantly enhanced PC-3 cell adhesion and invasiveness, while matrix stiffness emerged as a more potent driver of the metastatic phenotype (upregulated CD44 and vimentin expression) in PC-3 cells than the presence of CAFs, within the time frame of this study.

Overall, the proposed manufacturing pipeline offers a scalable, high-throughput platform for the study of prostate TME, which will be helpful for screening TME-targeting therapies and monitoring clinically relevant markers such as those associated with EMT and mechanobiology. Future refinements of this model (e.g., extended duration, increased cellular relevance) will further enhance its ability to predict clinical outcomes and obtain relevant NAMs to exploit therapeutic strategies. Ultimately, this work provides a robust foundation for developing patient-specific models to assess metastatic progression and response to therapeutic strategies, paving the way for advancements in personalized medicine.

## Supplementary Material



## Notes

Chemical characterization data for compounds (Figure S1: ^1^H NMR spectra and Figure S2: FT-IR); summary of mechanical properties (Table S1); pore size evaluation (Figure S3: SEM); optimization of cell culture media (Figure S4); microbead preparation (Figure S5: brightfield images and Figure S6: live/dead images); expression of EMT markers in PC-3 cells (Table S2 and Figure S7); PC-3 migration (Figure S8); and PC-3 invasion assay (Figure S9) (PDF)
