# Supplementary material for: Biofabricated Alginate Hydrogels to Study Prostate Tumoral Microenvironments In Vitro
Source: ACS Omega. 2026 Apr 29;11(18):26751–65. doi: 10.1021/acsomega.5c13436 (PMC13176988; doi:10.1021/acsomega.5c13436)
Supplement: Supplementary file 1 [file ao5c13436_si_001.pdf]

Biofabricated alginate hydrogels to study prostate tumoral micro-environments *in vitro*

Khalsa Al-Husaini[1,2], Eugenia Spessot[3], Esther Baena[4], Marco Domingos[5], Annalisa Tirella[1,3,*]

[1] Division of Pharmacy and Optometry, Medicine and Health, The University of Manchester, Manchester, UK

[2] Biology Department, College of Science, Sultan Qaboos University, Muscat, Oman

[3] Department of Industrial Engineering, Biotech Center for Biomedical Technologies, University of Trento, Trento, Italy

[4] Cancer Research UK Manchester Institute, The University of Manchester, Manchester, UK

[5] Department of Mechanical and Aerospace Engineering, School of Engineering, Faculty of Science and Engineering & Henry Royce Institute, The University of Manchester, Manchester, UK

[*] Corresponding author: annalisa.tirella@unitn.it

Supplementary Information

SI.1 Chemical characterization of sub products: ^{1}H-NMR

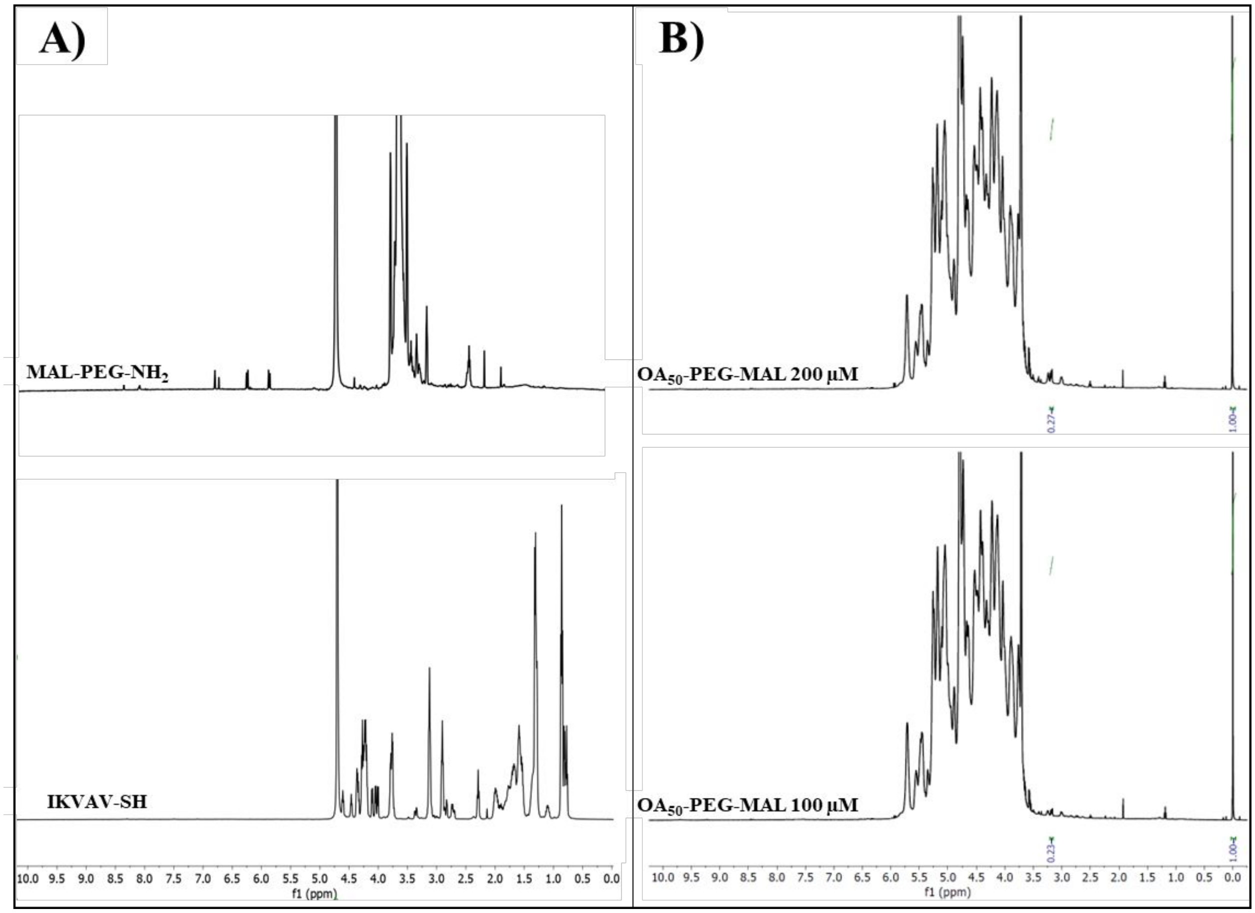

Figure SI.1. *^{1}H-NMR spectra of **A)** IKVAV-SH and MAL-PEG-NH$_2$ and **B)** OA$_{50}$-PEG-MAL.*

SI.2 Conjugation between oxidized Alginate (OA$_{50}$) and gelatin (G)

Aldehydes groups on OA$_{50}$ were quantified at a concentration of 6.5 μM/mg [1], and used to react with available ε-amino groups of lysine or hydroxylysine in gelatin (Schiff's base reaction). Gelatin FT-IR spectrum shows the characteristic bands of gelatin at 1626 cm^{-1} attributed to C=O stretching of amide I, and 1520 cm^{-1} of N-H deformation for amide Ⅱ. After mixing with alginate-based solutions (i.e. A$_0$ / OA$_{50}$, A$_0$ / OA$_{50}$-PEG-IKVAV), hydrogel precursor solutions or biomaterial inks are obtained. FT-IR spectra of hydrogel precursor solutions **A** and hydrogel precursor solutions **A-P** show the presence of the corresponding peaks of Schiff's base at around 1614-1617 cm^{-1} and 1536-1546 cm^{-1}. Schiff's base absorption band in hydrogel precursor solutions **A** is more pronounced probably due to the availability of more aldehyde groups to crosslink with ε-amino

groups of gelatin. The broader Schiff's base band at 1617 cm^{-1} in hydrogel precursor solutions **A-P** is possibly obtained because of overlapping with the band at 1626 $cm^{-1}$of amide I of un-crosslinked gelatin, as aldehyde groups are linked with laminin-mimicking peptides. Furthermore, the characteristic peak of gelatin at 1520 cm^{-1} representing amide II was not detected in both hydrogel formulas verifying the involvement of this group in the crosslinking reaction.

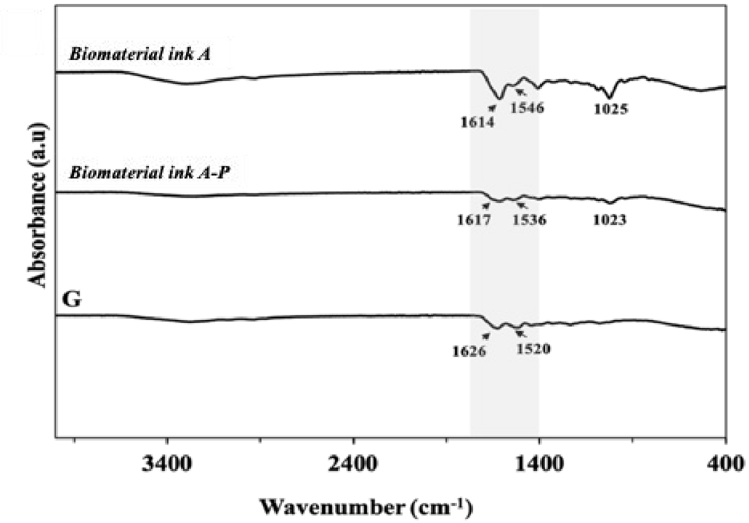

Figure SI.2. *FT-IR spectrum for gelatin (GEL) and biomaterial inks A and A-P (wavenumber range of 400-4000 cm^{-1}). Characteristic bands of gelatin were at 1626 cm^{-1} attributed to C=O stretching of amide I, and 1520 cm^{-1} of N-H deformation for amide Ⅱ.*

SI.3 Summary of mechanical properties of prostate-specific hydrogels

Table SI.1. Alginate-based hydrogels composition and mechanical properties. *Mechanical and rheological properties (i.e., Young's modulus E, storage modulus G', loss modulus G'', and loss tangent tan(d)) of obtained hydrogels. Alginate-hydrogels stiffness is proportional to crosslinking density, polymer concentration and molecular weight of polymers. Hydrogels were physically crosslinked using different concentration of $CaCl_2$ (i.e., 0.1 M, 0.3M) to modulate stiffness.*

**Hydrogel ID**	**Young's Modulus, E (Pa± SD)**	**Storage Modulus, G' (Pa mean ± SD)**	**Loss Modulus, G" (Pa mean ± SD)**	**Loss Tangent, tan(delta) (mean ± SD)**
A1	5593 ± 562	4143 ± 484	332 ± 31	0.080 ± 0.006
A3	12614 ± 1346	10293 ± 1457	1162 ± 190	0.113 ± 0.008
A1-P	3042 ± 521	1283 ± 422	141 ± 31	0.118 ± 0.026
A3-P	6598 ± 725	4151 ± 811	489 ± 105	0.111 ± 0.009

SI.4 Evaluation of pore size: Scanning Electron Microscope

To further corroborate porosity quantification, SEM micrographs were used to quantify pore size of hydrogels by using a field-emission scanning electron microscope (FE-SEM, Zeiss Supra 40, Carl Zeiss, Oberkochen, Germany). The hydrogels were freeze-dried and sputter coated with a thin layer of Pt/Pd before the analysis with the FE-SEM. The mean pore diameter was evaluated by using ImageJ software 2017 (NIH, Stapleton, NY, USA), measuring around 50 pore diameters for each of the tested conditions. Results were plotted as mean and st.dev in Figure SI.3.

The pore size of the hydrogels was found to be interconnected and in the range 50-800 um depending on the tested concentration.

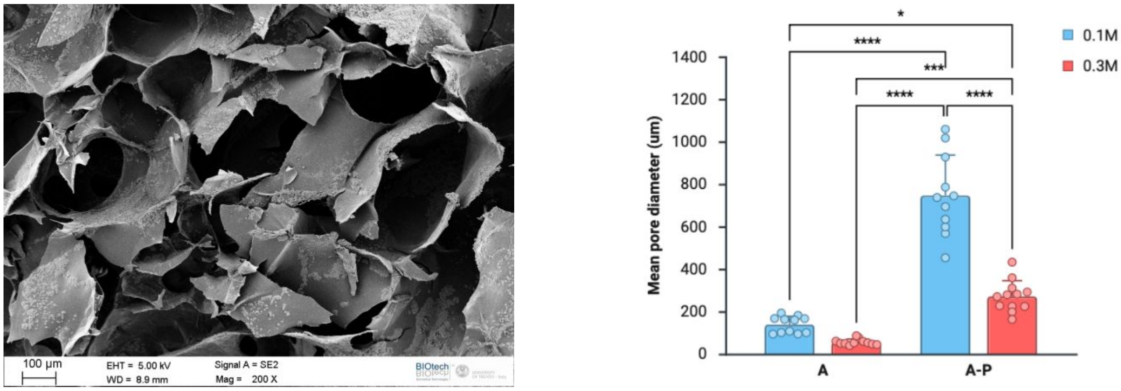

Figure SI.3. *Representative SEM image of A3-P hydrogel (left) and quantification of pore size using SEM micrograph. Data are represented as mean ± st.dev. of A1, A3, A1-P and A3-P (right).*

SI.5 Optimization of cell culture media for co-culture

PC-3 cells and hTERT PF179T CAFs were co-cultured using optimized conditions. For co-culture cells were maintained in F-12:EMEM medium (1:1 volume ratio). EMEM medium was used without L-glutamine and supplements were reduced 50% for all supplements detailed earlier for respective single cell culture. This selection of medium is optimized to maintain cell proliferation and viability for both cell lines **(Figure SI.4)**.

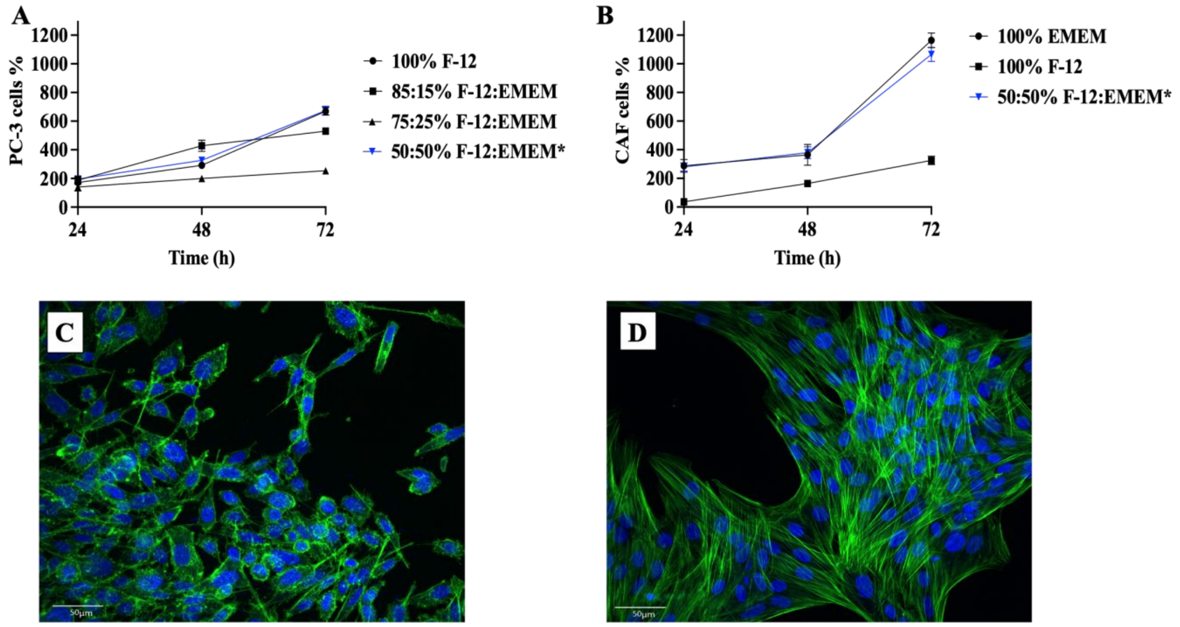

Figure SI.4. *Optimization of co-culture growth medium of co-culturing conditions varying F-12 and EMEM:* ***A)*** *PC-3 cells growth curves (complete F-12 used as control);* ***B)*** *hTERT PF179T CAF cells growth curves (complete EMEM used as control). Immunofluorescent images of:* ***C)*** *PC-3 and* ***D)*** *hTERT PF179T CAF cells cultured in optimized conditions (50:50% F-12:EMEM). Cells were stained with DAPI (nuclei, blue) and Phalloidin (F- Actin, green). Scale bars (50 µm).*

SI.6 Microbeads preparation and characterization for 3D prostate- and stroma-specific *in vitro* models

Brightfield images of 3D prostate and stroma *in vitro* models are shown in (**Figure SI.4**). Semi-quantitative assessment of PC-3 cell viability was performed using Live/Dead assay (**Figure SI.5**). A small percentage of dead cells was measured at day 1 (~20-30%), with approx. 75-85% of live cells measured after 1 week of culture, in all models and confirming the metabolic activity measured.

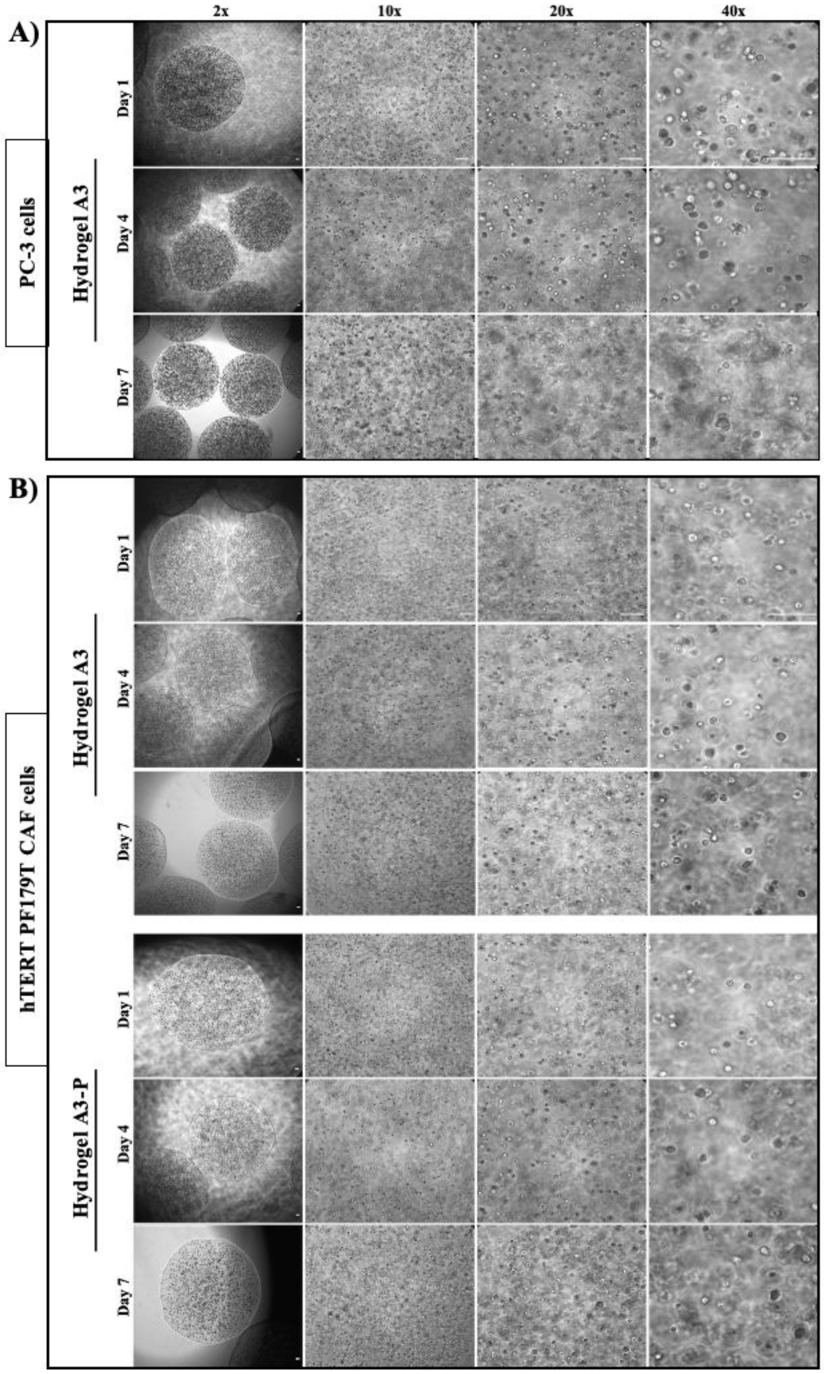

Figure SI.5. *Brightfield images of microbeads showing* ***A)*** *PC-3 prostate 3D in vitro models and* ***B)*** *Stroma-specific 3D in vitro models****.*** *Representative images of PC-3 cells encapsulated in hydrogel* ***A3****, showing formation of larger PC-3 cell aggregates in the latter. Images acquired with a 2×, 10×, 20×, and 40× objective respectively, at day1, 4, and 7. Scale bars 100 µm.*

Cells encapsulated in hydrogels **A1-P** and **A3-P** showed more uniformed and compact cellular aggregates compared to hydrogels **A1** and **A3**, indicating a higher level of reorganization and mobility within prostate-specific hydrogels at comparable stiffness in presence of PEG **(Figure SI.5)**.

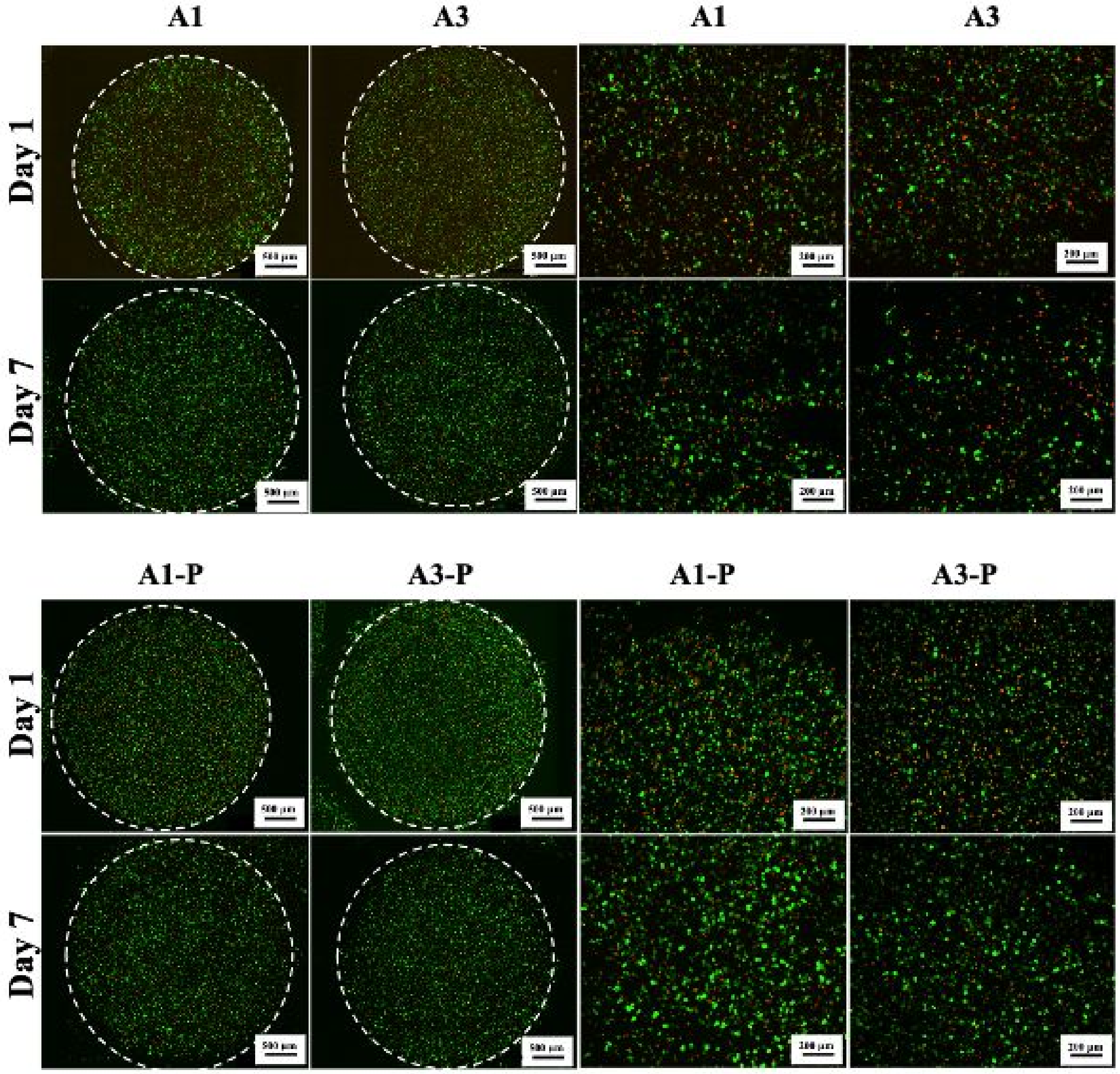

Figure SI.6. *Live/dead assay of PC-3 cells encapsulated in laminin-mimicking peptides enriched 3D in vitro models****.*** *Fluorescent images at different magnification show live proliferative cells (green cytoplasm) compared to dead cells (red nuclei) observed at different time points (i.e., day 3, day 7). Scale bars: 500 µm and 200 µm.*

SI.7 Expression of EMT markers in PC-3 cells

As control for 3D *in vitro* models, the expression of PCa progression and EMT was observed in PC-3 cells evaluating expression of selected markers, as reported in **Table SI.2**. To correlate expression of markers in different 3D *in vitro* prostate models with 2D *in vitro* models, PC-3 cells were analyzed after 3 days of culture on plastic (standard 2D *in vitro* models) as shown in **Figure SI.7**.

Table SI.2. *Flow cytometry markers and corresponding dilutions of isotype control, primary antibodies and secondary antibodies.*

Marker	Isotype control	Primary antibody	Secondary antibody
CD44	APC Rat IgG2b, κ Isotype Ctrl (400611, BioLegend)	APC anti-mouse/human CD44 Antibody (103012, BioLegend)	N/A
	(dilution 1:800)	Dilution (1:800)	
CD44v6	Mouse IgG1 Negative Control antibody (MCA928, Bio-Rad)	Mouse anti Human CD44v6 (MCA1730, Bio-Rad)	BV786 Rat Anti- Mouse IgG1, (742480, BD)
	(dilution 1:10)	(dilution 1:100)	(dilution 1:150)
Vimentin	Rat IgG2A APC Isotype control (IC006A, R&D systems)	APC Rat anti-human Vimentin (IC2105A, R&D systems)	N/A
	(dilution 1:10)	(dilution 1:20)	
E-cadherin	APC anti-human CD324 (B 263116, BioLegend)	APC Mouse IgG1 isotype (400121, BioLegend)	N/A
	(dilution 1:30)	(dilution 1:30)	

N/A = not applicable

Data were used as control to evaluate the impact of the 3D microenvironment on PC-3 phenotypes. The same protocol for staining and detection reported in the main manuscript was used.

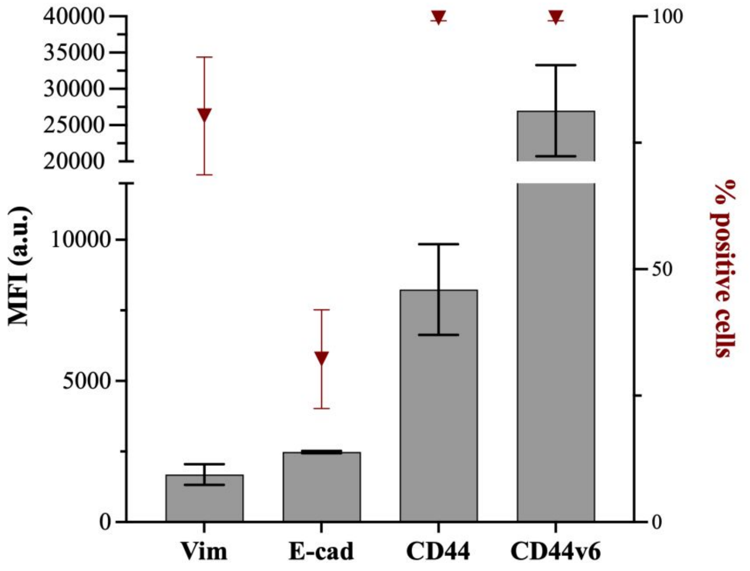

Figure SI.7. *Flow cytometry analysis in PC-3 cells cultured on 2D (control). Values are represented as mean and SD of N=3 independent experiments.*

SI.8 PC-3 migratory phenotype after conditioning in hydrogels enriched with laminin-mimicking peptides

PC-3 cells preconditioned (7 days) in *prostate-specific microbeads* were recovered and seeded in 24-well plate at a density of 1.5×10^5 cells/well and routinely cultured allowing cell adhesion (48 h, 37°C, 5% CO_2). A scratch was then performed in each well using a sterile 200 µL tip, cellular debris was removed by gentle washing with cell culture media to avoid cellular detachment. Cells were then cultured in low serum media (1% v/v FBS in F-12 with 1% v/v L-glutamine and 1% v/v PenStrep) to maintain low cell proliferation [42]. Brightfield images for scratch assay were acquired after 0, 24, and up to 120 h using an inverted microscope (Leica DMI6000, Leica Microsystems, UK). The area of scratch invaded by cells was calculated using ImageJ (v1.53a) by measuring the combined cellular area in the scratch over time.

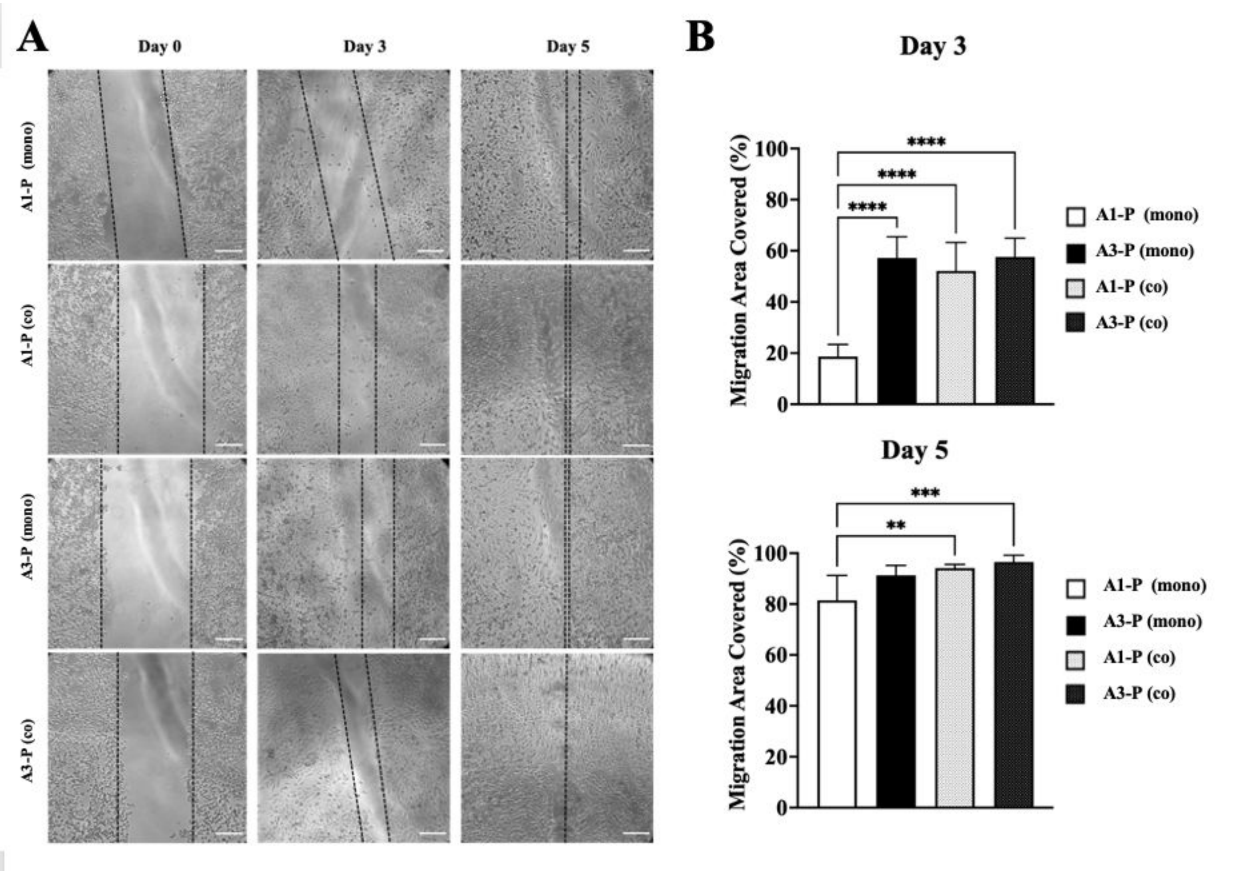

Figure SI.8. *Migration ability of PC-3 conditioned in laminin-mimicking peptides enriched 3D in vitro models: scratch assay.* ***A)*** *Brightfield images of the scratch closure. Scale bars 200 µm.* ***B)*** *Graphical representation of scratch area (%) covered by migratory PC-3 cells in µm^2, data is represented as mean and SD of N=2, n=2 independent experiments; P-values represented as *p ≤ 0.05, **p ≤ 0.01, ***p ≤ 0.001, ****p ≤ 0.0001).*

SI.9 Invasion assay

PC-3 cells preconditioned (7 days) in *prostate-specific microbeads* were recovered, stained with Cytopainter red by incubation with 1× dye diluted in cell culture media (1 h, 37°C, 5% CO_2). Cells were then washed trice with HBS and then embedded in a 1 mg/mL collagen hydrogel precursor diluted in 10× PBS and sterile water, adjusted to pH ~7.4 using sterile 1 M NaOH solution (aq.) and 7.5% NaHCO3 solution (aq.) following the supplier's instruction. Cell-laden hydrogels were routinely cultured (37°C, 5% CO_2) and the invasion imaged at day 0 and day 3 with confocal microscope using (Ex/Em 570/600 nm) filter to detect cytopainter (live cells, red).

To ensure monitoring of PC-3 cells preconditioned in engineered 3D prostate *in vitro* models, cells were stained (i.e., Cytopainter red) and calls invasion into collagen hydrogels monitored over time, following our previous study [2].

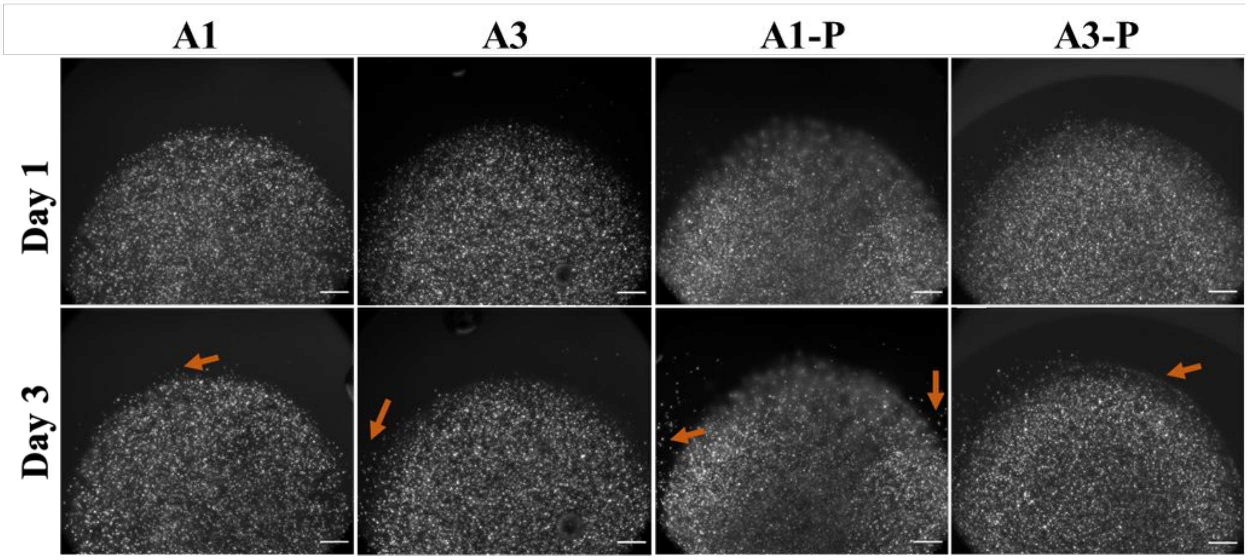

Figure SI.9. *Invasion ability of PC-3 conditioned in laminin-mimicking peptides enriched 3D in vitro models. Immunofluorescence images of Cytopainter red stained PC-3 cells then embedded in collagen hydrogel. Cells were imaged at day 0 and day 3 to evaluate invasion after pre-conditioning in different microenvironments. Scale bars 100 µm.*

References

[1] C. Zhao, A. Latif, K.J. Williams, A. Tirella, The characterization of molecular weight distribution and aggregation by asymmetrical flow field-flow fractionation of unmodified and oxidized alginate, Reactive and Functional Polymers 175 (2022) 105292. https://doi.org/10.1016/j.reactfunctpolym.2022.105292.

[2] L. Shah, A. Latif, K.J. Williams, A. Tirella, Role of stiffness and physico-chemical properties of tumour microenvironment on breast cancer cell stemness, Acta Biomaterialia 152 (2022) 273–289. https://doi.org/10.1016/j.actbio.2022.08.074.
